# The influence of bilingualism on gray matter volume in the course of aging: a longitudinal study

**DOI:** 10.3389/fnagi.2023.1193283

**Published:** 2023-07-20

**Authors:** Katharina Peitz, Johanna Stumme, Christiane Jockwitz, Nora Bittner, Svenja Caspers, Stefan Heim

**Affiliations:** ^1^Institute of Neuroscience and Medicine (INM-1), Research Center Jülich, Jülich, Germany; ^2^Department of Psychiatry, Psychotherapy and Psychosomatics, Medical Faculty, RWTH Aachen University, Aachen, Germany; ^3^Institute for Anatomy I, Medical Faculty and University Hospital Düsseldorf, Heinrich Heine University, Düsseldorf, Germany

**Keywords:** aging, bilingualism, inferior frontal gyrus, inferior parietal lobule, gray matter volume, brain reserve

## Abstract

**Background:**

Bilingualism is associated with higher gray matter volume (GMV) as a form of brain reserve in brain regions such as the inferior frontal gyrus (IFG) and the inferior parietal lobule (IPL). A recent cross-sectional study reported the age-related GMV decline in the left IFG and IPL to be steeper for bilinguals than for monolinguals. The present study aimed at supporting this finding for the first time with longitudinal data.

**Methods:**

In the current study, 200 participants aged 19 to 79 years (87 monolinguals, 113 sequential bilinguals, mostly native German speakers with variable second language background) were included. Trajectories of GMV decline in the bilateral IFG and IPL were analyzed in mono- and bilinguals over two time points (mean time interval: 3.6 years). For four regions of interest (left/right IFG and left/right IPL), mixed Analyses of Covariance were conducted to assess (i) GMV changes over time, (ii) GMV differences for language groups (monolinguals/bilinguals), and (iii) the interaction between time point and language group. Corresponding analyses were conducted for the two factors of GMV, surface area (SA) and cortical thickness (CT).

**Results:**

There was higher GMV in bilinguals compared to monolinguals in the IPL, but not IFG. While the left and right IFG and the right IPL displayed a similar GMV change in mono- and bilinguals, GMV decline within the left IPL was significantly steeper in bilinguals. There was greater SA in bilinguals in the bilateral IPL and a steeper CT decline in bilinguals within in the left IPL.

**Conclusion:**

The cross-sectional observations of a steeper GMV decline in bilinguals could be confirmed for the left IPL. Additionally, the higher GMV in bilinguals in the bilateral IPL may indicate that bilingualism contributes to brain reserve especially in posterior brain regions. SA appeared to contribute to bilinguals’ higher GMV in the bilateral IPL, while CT seemed to account for the steeper structural decline in bilinguals in the left IPL. The present findings demonstrate the importance of time as an additional factor when assessing the neuroprotective effects of bilingualism on structural features of the human brain.

## 1. Introduction

The process of aging is accompanied by an inter-individually variable decline in cognitive abilities (for reviews, cf. Hedden and Gabrieli, 2004; Grady, 2012; Salthouse, 2019) and brain structure (for reviews, cf. Bartrés-Faz and Arenaza-Urquijo, 2011; MacDonald and Pike, 2021). One of the protective factors potentially delaying age-related cognitive decline is bilingualism^1^ (e.g., Costumero et al., 2020; Zhang et al., 2020; Bialystok, 2021; Gallo et al., 2022). Bilingualism imposes unique challenges onto the human brain, such as a constant state of competition of the simultaneously active languages (e.g., Kroll et al., 2014; Bobb et al., 2020). Hence, bilinguals are required to engage in continuous conflict monitoring, conflict resolving, interference suppression of the non-target language and appropriate language switching (e.g., Bialystok, 1991; Green, 1998; Green and Abutalebi, 2013). The cognitive demands of bilingualism may depend on linguistic distance between languages spoken: It has been argued, for example, that typologically different languages may be more difficult to learn, while languages with similar typology might require greater inhibitory control when using one of the languages (Antoniou and Wright, 2017; for review, cf. Carthery-Goulart et al., 2023). Thus, both situations may result in considerable cognitive effort, but via contrasting mechanisms (cf. Danylkiv and Krafnick, 2020). Altogether, bilingualism may represent a form of cognitive exercise, which appears to induce a cognitive advantage also in terms of domain-general cognitive functions (for review, cf. Bialystok, 2017; Tao et al., 2021; but see also Paap and Greenberg, 2013; Antón et al., 2019).

The cognitive requirements of bilingualism have repercussions in brain structure. Bilingualism is usually associated with higher gray matter volume (GMV), higher cortical thickness (CT), and higher white matter integrity in regions related to language and domain-general control (for reviews, cf. e.g., Li et al., 2014; Hayakawa and Marian, 2019; Taylor et al., 2022). Among regions that have reliably shown higher GMV in bilinguals are the bilateral inferior frontal gyrus (IFG; Heim et al., 2019) and the bilateral inferior parietal lobule (IPL; Abutalebi et al., 2015; for reviews, cf. e.g., Li et al., 2014; Pliatsikas, 2020). Nonetheless, findings are variable between studies (cf. e.g., García-Pentón et al., 2016), and whole-brain analyses directly comparing mono- and bilinguals yield inconsistent results (meta-analysis in Danylkiv and Krafnick, 2020). This might, at least partially, result from a heterogeneity in samples and methodology (García-Pentón et al., 2016; Danylkiv and Krafnick, 2020). Additionally, the impact of bilingualism on brain structure appears to depend not only on the number of non-native languages spoken (Grogan et al., 2012), but also on experience-based factors such as age of acquisition (AoA), level of proficiency (LoP), amount of use of a second language, and frequency of language switching (cf. e.g., Li et al., 2014; DeLuca et al., 2019a,^2020^).

When investigating the effects of bilingualism on brain structure, one might distinguish simultaneous and sequential bilinguals from each other (Klein et al., 2014; Kaiser et al., 2015; for review, cf. e.g., Berken et al., 2017). These two groups differ in AoA, since simultaneous bilinguals learn two languages beginning from birth, while sequential bilinguals acquire a second language later in life. With respect to the context of language acquisition, simultaneous bilingualism usually corresponds to a rather naturalistic language experience, while sequential bilinguals often learn a second language in a classroom setting (Kaiser et al., 2015). Regarding structural brain adaptations to bilingualism, smaller cortical differences have been found for simultaneous than for sequential bilinguals when compared to monolinguals (Klein et al., 2014; for review, cf. Berken et al., 2017; Pliatsikas, 2020). Differences between simultaneous and sequential bilinguals might even persist into adulthood (Kaiser et al., 2015). Thus, it seems necessary to differentiate between these two forms of bilingualism when investigating the impact of bilingualism on brain structure. Altogether, bilingualism can be seen as a complex, heterogeneous, and dynamic experience, which might explain some of the discrepancies arising in bilingualism studies (cf. e.g., DeLuca et al., 2020).

Adaptations of the brain to bilingualism are supposed to counteract the effects of aging due to both, higher “cognitive reserve” and “brain reserve” in bilinguals (Bartrés-Faz and Arenaza-Urquijo, 2011). Cognitive reserve refers to differences in cognitive processing regarding efficiency, capacity, and flexibility of neural networks, including the ability for compensation when facing age-related structural atrophy (Stern, 2009; Stern et al., 2020). In bilinguals, an “anterior-to-posterior and subcortical shift” (BAPSS) has been described for task-induced neural activity with increasing bilingual experience (Grundy et al., 2017). As this shift is interpreted as increasing efficiency of cognitive processing in bilinguals, resulting from the cognitive requirements of bilingualism, BAPSS may represent a form of cognitive reserve in bilinguals (Grundy et al., 2017). When it comes to aging, however, a “posterior-to-anterior shift” (PASA) in neural activity has been observed (Davis et al., 2008). PASA is thought to correspond to a shift from automated to controlled processing. Therefore, it might represent a compensatory mechanism maintaining cognitive functioning despite the decline of brain structure in older adults (Davis et al., 2008). In bilinguals, who appear to use more posterior (and subcortical) brain regions for processing, as outlined in BAPSS, frontal brain regions may remain accessible for age-related compensation as described in PASA (Davis et al., 2008) to a greater extent than in monolinguals (Grundy et al., 2017), possibly reflecting another aspect of cognitive reserve in bilinguals.

Complementary to the concept of cognitive reserve, brain reserve refers to structural features such as brain volume, cell count, and number of synapses (Stern, 2009; Bartrés-Faz and Arenaza-Urquijo, 2011; Stern et al., 2020). It is assumed that individuals with higher brain reserve can tolerate more decline before reaching a certain threshold under which clinical deficits become evident (Stern, 2009; Bartrés-Faz and Arenaza-Urquijo, 2011; Stern et al., 2020). Interestingly, the structural adaptations of the brain to bilingualism appear to result in higher brain reserve, as higher gray matter volume (GMV), higher cortical thickness (CT), and higher white matter integrity found in bilinguals when compared to monolinguals (for reviews, cf. e.g., Li et al., 2014; Hayakawa and Marian, 2019; Taylor et al., 2022) can be seen as proxies of brain reserve. When combining the two concepts, higher brain reserve as well as cognitive reserve in bilinguals can explain how bilingualism may delay age-related cognitive decline not only in healthy older subjects (e.g., Bialystok et al., 2004; Gold et al., 2013; Bak et al., 2014), but also in terms of neurodegenerative diseases (i.e., bilingualism appears to delay the clinical onset of dementia by four to five years; Craik et al., 2010; Alladi et al., 2013; Perani et al., 2017; meta-analyses in Anderson et al., 2020; Paulavicius et al., 2020; see also Voits et al., 2020).

A concept closely related to brain reserve is “brain maintenance.” Greater brain maintenance corresponds to reduced age-related structural decline over time, possibly modulated by lifestyle or genetic factors (Stern et al., 2020). While brain reserve corresponds to brain structure at a single time point, brain maintenance is best evaluated longitudinally (Stern et al., 2020). Recently, Costumero et al. (2020) found less GMV decline in bilinguals compared to monolinguals across a time interval of 7 months (all individuals being older adults with diagnosis of mild cognitive impairment), which could be interpreted as first evidence for brain maintenance in bilinguals. However, additional longitudinal studies investigating trajectories of structural change in the older bilingual brain are needed to further investigate the relationship between brain maintenance and bilingualism.

Interestingly, a recent cross-sectional study found evidence not only for higher GMV, but also for a steeper GMV decline with aging in the left IFG and IPL in bilinguals compared to monolinguals (Heim et al., 2019). The left IFG is described as a critical brain region for language production and comprehension, e.g., in terms of lexical retrieval, semantic and phonological fluency, and syntax processing (Heim et al., 2009; for review, cf. Friederici, 2011; Li et al., 2014). In bilinguals, the left IFG seems to be involved in response selection, e.g., in language switching tasks (for review, cf. Abutalebi and Green, 2016). The left IPL, on the other hand, is involved in phonological as well as semantic processing (for review, cf. Li et al., 2014; Binkofski et al., 2016) and has been found to play a key role in second language acquisition (Barbeau et al., 2017) and vocabulary knowledge (Lee et al., 2007). Their role in language processing might explain why these two brain regions show structural differences, such as higher GMV, in bilinguals when compared to monolinguals. However, with evidence for a steeper GMV decline in the left IFG and IPL in bilinguals, volume differences between mono- and bilinguals appear to diminish over time, with a higher persistence of a bilingual brain reserve in posterior than anterior brain regions (Heim et al., 2019). This pattern might reflect a more pronounced activation of posterior brain regions in bilinguals compared to monolinguals, as described in BAPSS (Grundy et al., 2017). Nevertheless, since cross-sectional results may differ substantially from results obtained from longitudinal data (e.g., Hedden and Gabrieli, 2004; Salthouse, 2010), the differential GMV trajectories in monolinguals and bilinguals predicted by cross-sectional studies remain to be confirmed in longitudinal analyses.

Therefore, the present study was devised as follows: (1) The previous cross-sectional study (Heim et al., 2019) was to be replicated in a large-scale population-based longitudinal design over two time points. Hence, trajectories of GMV decline over time were investigated in mono- and bilinguals in the cytoarchitectonically defined IFG (Amunts et al., 1999) and IPL (Caspers et al., 2006, 2008). We predicted higher GMV in bilinguals in the IFG and IPL in both hemispheres. Additionally, we expected a steeper GMV decline in bilinguals in the IFG (cf. Heim et al., 2019) and IPL (cf. Abutalebi et al., 2015) in the left, but not necessarily in the right hemisphere. (2) In a refined model, age, sex, education, and intracranial volume (ICV) were included as covariates. (3) To set a focus on the investigation of the older adult population, basic as well as refined analyses were conducted using a subsample, comprising only participants ≥ 55 years old. (4) Finally, regression analyses were conducted to evaluate the influence of experience-based factors such as AoA, LoP, and number of languages actively spoken, on GMV in the IFG and IPL in bilinguals.

## 2. Materials and methods

### 2.1. Participants

The current sample was derived from the longitudinal population-based 1000BRAINS study (Caspers et al., 2014). 1000BRAINS aims at investigating inter-individual variability in brain aging in healthy adults. Subjects for 1000BRAINS were drawn from the Heinz-Nixdorf Recall (HNR) study and the subsequent HNR MultiGeneration study, which have been conducted in the German Ruhr area to assess risk factors for atherosclerotic disease, myocardial infarction and cardiac death (Schmermund et al., 2002; Erbel et al., 2012). With 1000BRAINS being a population-based study, exclusion was based solely on contraindications to magnetic resonance imaging, i.e., coronary artery stents, cardiac pacemakers, surgical implants or prostheses in the trunk or head, claustrophobia, a history of neurosurgery, the presence of tattoos or permanent make-up on the head, and dental implants and bridges (the latter being a relative contraindication; see Caspers et al., 2014). Written informed consent was obtained from all subjects prior to participation in 1000BRAINS. The study was performed in accordance with the Declaration of Helsinki. All methods were approved by the local Ethics Committee of the University of Essen, Germany.

From the initial 1000BRAINS cohort (*n* = 1,314), 466 subjects took part in a second examination. From this sample, 269 individuals completed the Language Experience and Proficiency Questionnaire (LEAP-Q; Marian et al., 2007) and had structural MRI data sets from two time points and were thus eligible for the current longitudinal study. Left-handed individuals (*n* = 8, Laterality Quotient < −60 as assessed with the Edinburgh Handedness Inventory; Oldfield, 1971) or individuals who did not provide any data regarding their handedness (*n* = 1) were excluded. Further exclusion criteria based on LEAP-Q data were simultaneous bilingualism (*n* = 6; see discussion above for putative structural differences between simultaneous and sequential bilinguals; for reviews, cf. Berken et al., 2017; Pliatsikas, 2020) and developmental first language deficiencies in any modality (speaking, comprehending, reading, writing) (*n* = 32). Moreover, eight subjects had to be excluded due to methodological problems within the preprocessing of structural brain images. Further exclusion of 14 participants due to outlier correction (GMV, CT and/or white matter surface area (SA) exceeding three standard deviations from the mean) resulted in the final sample of 200 participants (87 monolinguals and 113 bilinguals; Table 1). For analyses of the older subsample, 154 subjects could be included (83 monolinguals and 71 bilinguals; Table 1).

**TABLE 1:** Study sample: demographic characteristics.

	Total sample (*n* = 200)		Subsample (participants ≥ 55 y, *n* = 154)	
	Monolinguals (*n* = 87)	Bilinguals (*n* = 113)	Monolinguals (*n* = 83)	Bilinguals (*n* = 71)
**Gender**				
% Female	44.8	41.6	44.6	38.0
% Male	55.2	58.4	55.4	62.0
**Age at t1 (years)**				
Mean (SD)	66.4 (7.7)	56.7 (12.6)	67.2 (6.4)	64.7 (6.2)
Minimum	33.7	18.5	56.2	55.1
Maximum	79.4	78.4	79.4	78.4
Education level at t1 (SD)	6.0 (1.8)	7.6 (1.7)	6.0 (1.8)	7.6 (1.9)
Time interval between t1 and t2 (SD) (years)	3.8 (0.8)	3.4 (0.8)	3.8 (0.8)	3.6 (0.8)

Group characteristics for monolinguals and bilinguals for the total sample (*n* = 200) as well as for the subsample including only participants ≥ 55 years old (*n* = 154). Key: t1, first time point; t2, second time point; SD, standard deviation; y, years.

### 2.2. Assessment of bilingualism

Participants’ second language status was determined using LEAP-Q data (Marian et al., 2007) during the first examination. The LEAP-Q is a questionnaire to set up language profiles in bilinguals and multilinguals regarding age of acquisition, proficiency of all modalities, manner of acquisition and immersion in a bilingual environment. For the present study, individuals who indicated to currently speak, understand, read and/or write in more than one language were classified as bilinguals. Consequently, participants with no or lost second language abilities were classified as monolinguals. While monolinguals within the current sample spoke German only, language backgrounds for bilinguals (mostly native German speakers) are reported in Table 2.

**TABLE 2:** Languages spoken among bilinguals included in the present study.

Language	Total sample	Subsample
	Bilinguals (*n* = 113) with language abilities (%)	Bilinguals (*n* = 71) with language abilities (%)
German	100.00	100.00
English	97.35	95.77
French	39.82	42.25
Spanish	12.39	12.68
Latin	9.73	7.04
Italian	6.19	5.63
Dutch	3.54	5.63
Russian	3.54	5.63
Polish	2.65	4.23
Other	5.31	5.64

Distribution of bilinguals who reported language abilities for the respective language in percent. “Other” includes: Ancient Greek, Finnish, Portuguese, Serbian, Swedish, Ukrainian. Dialects were not considered within the present study, since only one participant reported language abilities for a dialect, and this participant had to be excluded from the sample due to simultaneous bilingualism.

Of the bilinguals, 20.4% reported a very good and 46.0% a good level of proficiency for speaking, understanding, reading and/or writing in their second language, while 26.5% reported an adequate and 7.1% a low level of proficiency as maximum. Second languages were rated according to self-reported proficiency in the respective language. The mean age of acquisition of the second language that was associated with the highest proficiency was 13.2 years (± 7.2 years).

### 2.3. MRI data

#### 2.3.1. Data acquisition

Magnetic resonance imaging data were acquired at two time points (t1 and t2, mean time interval ± SD: 3.6 ± 0.8 years) on a 3T Siemens Tim-TRIO MR scanner (Erlangen, Germany). 3D high-resolution T1-weighted magnetization-prepared rapid acquisition gradient-echo (MPRAGE) scans were obtained for each participant as part of the whole imaging protocol (for further details, see Caspers et al., 2014) at each time point using a 32-channel head coil (176 slices, slice thickness = 1 mm, repetition time = 2,250 ms, echo time = 3.03 ms, field of view = 256 × 256 mm^2^, flip angle = 9°, voxel resolution = 1 × 1 × 1 mm^3^).

#### 2.3.2. Image processing

Magnetic resonance imaging sequences were processed using the automated surface-based longitudinal pipeline implemented in FreeSurfer 6.0 (for a detailed description, see Reuter et al., 2012), which consists of three major steps: First, structural images from both time points were processed individually, corresponding to the processing of cross-sectional data (cf. Dale et al., 1999; Fischl et al., 1999a). Second, a within-subject template was built across the resulting data from the two time points (Reuter et al., 2012). Third, information from both, the cross-sectional as well as longitudinal preprocessing, were used to generate surface maps for GMV, CT, and SA (Reuter et al., 2012).

**FIGURE 1:**
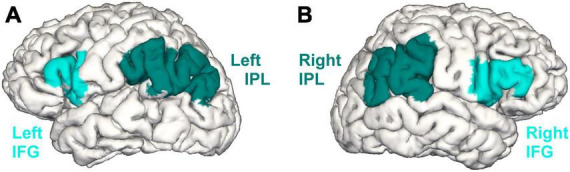
The inferior frontal gyrus (IFG) and the inferior parietal lobule (IPL) in the left **(A)** and right **(B)** hemisphere based on the cytoarchitectonic probabilistic Jülich-Brain atlas (Amunts et al., 2020).

The present study targeted two language-relevant regions of interest (ROIs): the left IFG (Amunts et al., 1999) and the left IPL (Caspers et al., 2006, 2008; Figure 1A). The right IFG and right IPL were analyzed as control regions (Figure 1B). Predefined ROI masks derived from the probabilistic cytoarchitectonic Jülich-Brain atlas (Amunts et al., 2020) were mapped onto the reconstructed surface maps. The masks were inspected by a neuroanatomy specialist (S.C.) when overlayed on FreeSurfer’s fsaverage template (Fischl et al., 1999b) and manually corrected when necessary. For both ROIs and control regions, GMV as well as CT and SA were extracted from the longitudinally processed data.

Corresponding to the cross-sectional analyses by Heim et al. (2019), the current study focused on the analysis of GMV. GMV, as the product of CT and SA, can be seen as a multi-determined parameter that may provide insights into structural variability of the brain that might not be captured by sole analyses of CT or SA (Nicolaisen-Sobesky et al., 2022). However, since CT and SA are thought to be genetically and phenotypically independent from each other (Panizzon et al., 2009; Winkler et al., 2010), CT and SA analyses are reported as well, providing a first step to disentangling the picture of age-related structural change in the bilingual brain.

### 2.4. Statistical analysis

Statistical analysis of the extracted values was performed with the IBM Statistical Package for Social Sciences (SPSS), version 27.0.0^2^.

#### 2.4.1. Total sample

##### 2.4.1.1. Basic ANCOVA models

To evaluate whether bilinguals show a steeper GMV decline when compared to monolinguals, mixed Analyses of Covariance (ANCOVAs) were conducted separately for each of the four ROIs (left/right IFG and left/right IPL). As the aim of the present study was to replicate the previous cross-sectional analysis (Heim et al., 2019) in a longitudinal design, the basic ANCOVA model was designed as similar to the cross-sectional study as possible. Therefore, GMV values from both time points were treated as dependent variables, while language group (monolinguals/bilinguals) and age group (younger/older participants, sample split at the age median of 62.8 years; for demographic information, see Supplementary Table 1) were included as between-subject factors (to further relate the findings from the present longitudinal study to the former cross-sectional analysis, see Supplementary material: Supplementary methods and Supplementary Table 2). Additionally, in the present study, time point (t1/t2) was included as within-subject factor, while time interval between the two measurements was treated as covariate of no interest. The resulting 2 × 2 × 2 ANCOVA allowed the assessment of (i) GMV changes over time between t1 and t2, (ii) differences in GMV for language groups and age groups, and (iii) the interaction between time point and language group.

To assess putative interhemispheric differences regarding GMV trajectories over time in bilinguals compared to monolinguals in the IFG and IPL, hemisphere (left/right) was included in an additional ANCOVA model as within-subject factor. This resulted in a 2 × 2 × 2 × 2 ANCOVA with time interval as covariate. All analyses were additionally performed for CT and SA.

##### 2.4.1.2. Refined ANCOVA models

Following the mere replication of the cross-sectional study (Heim et al., 2019) in a design over two time points, the ANCOVA models were refined in a next step. To control for the effects of potential confounds on GMV, age, sex, education (as assessed with the International Standard Classification of Education; UNESCO Institute for Statistics [UIS], 2012), and intracranial volume (ICV) were included into the basic ANCOVA model as covariates of no interest. Due to the inclusion of age as a covariate, age group was excluded as between-subject factor, resulting in a 2 × 2 ANCOVA with GMV values from both time points as dependent variables, language group as between-subject factor, time point as within-subject factor, and age, sex, education, ICV, and time interval as covariates. In a next step, hemisphere was added to the model as within-subject factor. Analogous analyses were performed for CT and SA.

One may discuss whether ICV can be seen as a meaningful covariate for CT and SA analyses (for an investigation of the relationship between ICV and GMV, CT, and SA, see Im et al., 2008). Thus, additional CT and SA analyses only including age, sex, education, and time interval as covariates were conducted. For the sake of comparison, corresponding GMV analyses were performed as well.

##### 2.4.1.3. Regression analyses

To evaluate the influence of experience-based factors such as AoA, LoP, and number of actively spoken languages on GMV in the bilateral IFG and IPL in bilinguals, regression analyses were conducted. Since the experience-based factors were, by definition, available for bilinguals only, monolinguals were excluded from the models. For each of the four ROIs, three separate analyses were performed with (1) GMV at t1, (2) GMV at t2, and (3) GMV differences between t1 and t2 as dependent variable, respectively. Variables of interest (AoA, LoP, and number of actively spoken languages) and nuisance variables (age, sex (males set to 0, females to 1), education, ICV) were treated as predictors in all models. For the analyses of GMV differences between t1 and t2, time interval was added as additional nuisance variable. Corresponding analyses were performed for CT and SA. Again, additional analyses not including ICV as a nuisance variable were conducted as well.

#### 2.4.2. Subsample analyses: participants ≥ 55 years old

Within the present study, participants’ age ranged from 18.5 to 79.4 years at time point t1. To set a focus on the investigation of the inter-individual variability within an older adult population with a more homogenous distribution of mono- and bilinguals, mixed ANCOVAs and regression analyses were not only conducted with the total sample of 200 participants, but also with a subsample comprising only subjects ≥ 55 years of age at t1 (83 monolinguals, 71 bilinguals, Table 1).

For the subsample, basic and refined ANCOVAs as well as regression analyses were performed corresponding to GMV analyses of the total sample. The only difference was the exclusion of age group as between-subject factor in the basic ANCOVA model, since only older adults were investigated here. The same analyses were conducted for CT and SA.

## 3. Results

### 3.1. ANCOVA models

In the next section, results for basic and refined ANCOVA models (the latter including ICV as a covariate) are presented (see also Tables 3–8). Results for refined models excluding ICV as a covariate show a similar pattern to analyses including ICV and are reported in Supplementary Tables 3–5.

**TABLE 3:** Results for basic mixed ANCOVA models for values of GMV, CT, and SA from two time points for 200 participants.

	Left hemisphere			Right hemisphere		
	Language group	Age group	Time point × Language group	Language group	Age group	Time point × Language group
**GMV**						
IFG	*F*(1,195) = 0.007 *p* = 0.933 partial η^2^ < 0.001	*F*(1,195) = 13.696 *p* < 0.001 *** partial η^2^ = 0.066	*F*(1,195) = 0.001 *p* = 0.976 partial η^2^ < 0.001	*F*(1,195) = 2.968 *p* = 0.087 partial η^2^ = 0.015	*F*(1,195) = 7.815 *p* = 0.006 ** partial η^2^ = 0.039	*F*(1,195) = 0.379 *p* = 0.539 partial η^2^ = 0.002
IPL	*F*(1,195) = 9.966 *p* = 0.002 ** partial η^2^ = 0.049	*F*(1,195) = 6.071 *p* = 0.015 * partial η^2^ = 0.030	*F*(1,195) = 4.211 *p* = 0.042 * partial η^2^ = 0.021	*F*(1,195) = 8.340 *p* = 0.004 ** partial η^2^ = 0.041	*F*(1,195) = 12.623 *p* < 0.001 *** partial η^2^ = 0.061	*F*(1,195) = 0.717 *p* = 0.398 partial η^2^ = 0.004
**CT**						
IFG	*F*(1,195) = 1.171 *p* = 0.280 partial η^2^ = 0.006	*F*(1,195) = 13.234 *p* < 0.001 *** partial η^2^ = 0.064	*F*(1,195) = 1.840 *p* = 0.176 partial η^2^ = 0.009	*F*(1,195) = 0.025 *p* = 0.873 partial η^2^ < 0.001	*F*(1,195) = 17.583 *p* < 0.001 *** partial η^2^ = 0.083	*F*(1,195) = 0.093 *p* = 0.760 partial η^2^ < 0.001
IPL	*F*(1,195) = 1.299 *p* = 0.256 partial η^2^ = 0.007	*F*(1,195) = 33.953 *p* < 0.001 *** partial η^2^ = 0.148	*F*(1,195) = 6.653 *p* = 0.011 * partial η^2^ = 0.033	*F*(1,195) = 0.061 *p* = 0.806 partial η^2^ < 0.001	*F*(1,195) = 27.377 *p* < 0.001 *** partial η^2^ = 0.123	*F*(1,195) = 0.891 *p* = 0.346 partial η^2^ = 0.005
**SA**						
IFG	*F*(1,195) = 0.565 *p* = 0.453 partial η^2^ = 0.003	*F*(1,195) = 3.772 *p* = 0.054 partial η^2^ = 0.019	*F*(1,195) = 1.865 *p* = 0.174 partial η^2^ = 0.009	*F*(1,195) = 2.619 *p* = 0.107 partial η^2^ = 0.013	*F*(1,195) = 1.487 *p* = 0.224 partial η^2^ = 0.008	*F*(1,195) = 0.230 *p* = 0.632 partial η^2^ = 0.001
IPL	*F*(1,195) = 7.924 *p* = 0.005 ** partial η^2^ = 0.039	*F*(1,195) = 0.076 *p* = 0.783 partial η^2^ < 0.001	*F*(1,195) = 1.135 *p* = 0.288 partial η^2^ = 0.006	*F*(1,195) = 7.987 *p* = 0.005 ** partial η^2^ = 0.039	*F*(1,195) = 1.055 *p* = 0.306 partial η^2^ = 0.005	*F*(1,195) = 0.516 *p* = 0.474 partial η^2^ = 0.003

*p < 0.05; **p < 0.01; ***p < 0.001.

Key: GMV, gray matter volume; CT, cortical thickness; SA, surface area; IFG, inferior frontal gyrus; IPL, inferior parietal lobule; ANCOVA, Analysis of Covariance.

**TABLE 4:** Results for refined mixed ANCOVA models for values of GMV, CT, and SA from two time points for 200 participants, including age, sex, education, and ICV as further covariates.

	Left hemisphere		Right hemisphere	
	Language group	Time point × Language group	Language group	Time point × Language group
**GMV**				
IFG	*F*(1,193) = 1.253 *p* = 0.264 partial η^2^ = 0.006	*F*(1,193) = 0.061 *p* = 0.806 partial η^2^ < 0.001	*F*(1,193) < 0.001 *p* = 0.984 partial η^2^ < 0.001	*F*(1,193) = 0.570 *p* = 0.451 partial η^2^ = 0.003
IPL	*F*(1,193) = 2.376 *p* = 0.125 partial η^2^ = 0.012	*F*(1,193) = 3.475 *p* = 0.064 partial η^2^ = 0.018	*F*(1,193) = 4.110 *p* = 0.044 * partial η^2^ = 0.021	*F*(1,193) = 0.508 *p* = 0.477 partial η^2^ = 0.003
**CT**				
IFG	*F*(1,193) = 0.001 *p* = 0.972 partial η^2^ < 0.001	*F*(1,193) = 0.258 *p* = 0.612 partial η^2^ = 0.001	*F*(1,193) = 1.224 *p* = 0.270 partial η^2^ = 0.006	*F*(1,193) = 0.211 *p* = 0.647 partial η^2^ = 0.001
IPL	*F*(1,193) = 0.530 *p* = 0.468 partial η^2^ = 0.003	*F*(1,193) = 4.700 *p* = 0.031 * partial η^2^ = 0.024	*F*(1,193) = 1.045 *p* = 0.308 partial η^2^ = 0.005	*F*(1,193) = 0.046 *p* = 0.831 partial η^2^ < 0.001
**SA**				
IFG	*F*(1,193) = 1.670 *p* = 0.198 partial η^2^ = 0.009	*F*(1,193) = 0.516 *p* = 0.474 partial η^2^ = 0.003	*F*(1,193) = 0.213 *p* = 0.645 partial η^2^ = 0.001	*F*(1,193) = 0.136 *p* = 0.713 partial η^2^ = 0.001
IPL	*F*(1,193) = 1.277 *p* = 0.260 partial η^2^ = 0.007	*F*(1,193) = 0.261 *p* = 0.610 partial η^2^ = 0.001	*F*(1,193) = 7.493 *p* = 0.007 ** partial η^2^ = 0.037	*F*(1,193) = 0.145 *p* = 0.703 partial η^2^ = 0.001

*p < 0.05; **p < 0.01.

Key: GMV, gray matter volume; CT, cortical thickness; SA, surface area; IFG, inferior frontal gyrus; IPL, inferior parietal lobule; ICV, intracranial volume; ANCOVA, Analysis of Covariance.

**TABLE 5:** Results for basic mixed ANCOVA models for values of GMV, CT, and SA from two time points for the subsample of 154 participants.

	Left hemisphere		Right hemisphere	
	Language group	Time point × Language group	Language group	Time point × Language group
**GMV**				
IFG	*F*(1,151) = 0.184 *P* = 0.669 partial η^2^ = 0.001	*F*(1,151) = 0.380 *p* = 0.539 partial η^2^ = 0.003	*F*(1, 151) = 0.866 *p* = 0.354 partial η^2^ = 0.006	*F*(1,151) = 0.199 *p* = 0.656 partial η^2^ = 0.001
IPL	*F*(1,151) = 8.703 *p* = 0.004 ** partial η^2^ = 0.054	*F*(1,151) = 2.287 *p* = 0.133 partial η^2^ = 0.015	*F*(1,151) = 8.655 *p* = 0.004 ** partial η^2^ = 0.054	*F*(1,151) = 0.111 *p* = 0.740 partial η^2^ = 0.001
**CT**				
IFG	*F*(1,151) = 0.715 *p* = 0.399 partial η^2^ = 0.005	*F*(1,151) = 0.023 *p* = 0.880 partial η^2^ < 0.001	*F*(1,151) < 0.001 *p* = 0.994 partial η^2^ < 0.001	*F*(1,151) < 0.001 *p* = 0.991 partial η^2^ < 0.001
IPL	*F*(1,151) = 0.691 *p* = 0.407 partial η^2^ = 0.005	*F*(1,151) = 3.244 *p* = 0.074 partial η^2^ = 0.021	*F*(1,151) = 0.002 *p* = 0.965 partial η^2^ < 0.001	*F*(1,151) = 0.080 *p* = 0.777 partial η^2^ = 0.001
**SA**				
IFG	*F*(1,151) = 0.790 *p* = 0.375 partial η^2^ = 0.005	*F*(1,151) = 0.189 *p* = 0.665 partial η^2^ = 0.001	*F*(1,151) = 1.047 *p* = 0.308 partial η^2^ = 0.007	*F*(1,151) = 0.415 *p* = 0.520 partial η^2^ = 0.003
IPL	*F*(1,151) = 7.191 *p* = 0.008 ** partial η^2^ = 0.045	*F*(1,151) = 0.268 *p* = 0.606 partial η^2^ = 0.002	*F*(1,151) = 9.211 *p* = 0.003 ** partial η^2^ = 0.057	*F*(1,151) = 0.010 *p* = 0.921 partial η^2^ < 0.001

**p < 0.01.

Key: GMV, gray matter volume; CT, cortical thickness; SA, surface area; IFG, inferior frontal gyrus; IPL, inferior parietal lobule; ANCOVA, Analysis of Covariance.

**TABLE 6:** Results for refined mixed ANCOVA models for values of GMV, CT, and SA from two time points for the subsample of 154 participants, including age, sex, education, and ICV as further covariates.

	Left hemisphere		Right hemisphere	
	Language group	Time point × Language group	Language group	Time point × Language group
**GMV**				
IFG	*F*(1,147) = 0.963 *p* = 0.328 partial η^2^ = 0.007	*F*(1,147) = 0.153 *p* = 0.696 partial η^2^ = 0.001	*F*(1,147) = 0.011 *p* = 0.917 partial η^2^ < 0.001	*F*(1,147) = 0.467 *p* = 0.496 partial η^2^ = 0.003
IPL	*F*(1,147) = 3.517 *p* = 0.063 partial η^2^ = 0.023	*F*(1,147) = 2.875 *p* = 0.092 partial η^2^ = 0.019	*F*(1,147) = 5.100 *p* = 0.025 * partial η^2^ = 0.034	*F*(1,147) = 1.237 *p* = 0.268 partial η^2^ = 0.008
**CT**				
IFG	*F*(1,147) = 0.009 *p* = 0.924 partial η^2^ < 0.001	*F*(1,147) = 0.097 *p* = 0.755 partial η^2^ = 0.001	*F*(1,147) = 1.782 *p* = 0.184 partial η^2^ = 0.012	*F*(1,147) = 0.011 *p* = 0.917 partial η^2^ < 0.001
IPL	*F*(1,147) = 0.528 *p* = 0.468 partial η^2^ = 0.004	*F*(1,147) = 4.118 *p* = 0.044 * partial η^2^ = 0.027	*F*(1,147) = 0.464 *p* = 0.497 partial η^2^ = 0.003	*F*(1,147) = 0.200 *p* = 0.655 partial η^2^ = 0.001
**SA**				
IFG	*F*(1,147) = 1.522 *p* = 0.219 partial η^2^ = 0.010	*F*(1,147) = 0.405 *p* = 0.526 partial η^2^ = 0.003	*F*(1,147) = 0.499 *p* = 0.481 partial η^2^ = 0.003	*F*(1,147) = 0.483 *p* = 0.488 partial η^2^ = 0.003
IPL	*F*(1,147) = 1.913 *p* = 0.169 partial η^2^ = 0.013	*F*(1,147) = 0.092 *p* = 0.762 partial η^2^ = 0.001	*F*(1,147) = 7.526 *p* = 0.007 ** partial η^2^ = 0.049	*F*(1,147) = 0.355 *p* = 0.552 partial η^2^ = 0.002

*p < 0.05; **p < 0.01.

Key: GMV, gray matter volume; CT, cortical thickness; SA, surface area; IFG, inferior frontal gyrus; IPL, inferior parietal lobule; ICV, intracranial volume; ANCOVA, Analysis of Covariance.

**TABLE 7:** Results for mixed ANCOVA models including hemisphere as within-subject factor for values of GMV, CT, and SA from two time points for 200 participants.

	Basic ANCOVA model		Refined ANCOVA model	
	Hemisphere	Hemisphere × Time point × Language group	Hemisphere	Hemisphere × Time point × Language group
**GMV**				
IFG	*F*(1,195) = 2.144 *p* = 0.145 partial η^2^ = 0.011	*F*(1,195) = 0.265 *p* = 0.607 partial η^2^ = 0.001	*F*(1,193) = 0.243 *p* = 0.623 partial η^2^ = 0.001	*F*(1,193) = 0.134 *p* = 0.715 partial η^2^ = 0.001
IPL	*F*(1,195) = 14.356 *p* < 0.001 *** (L > R) partial η^2^ = 0.069	*F*(1,195) = 2.490 *p* = 0.116 partial η^2^ = 0.013	*F*(1,193) = 0.001 *p* = 0.980 partial η^2^ < 0.001	*F*(1,193) = 2.185 *p* = 0.141 partial η^2^ = 0.011
**CT**				
IFG	*F*(1,195) = 7.867 *p* = 0.006 ** (L > R) partial η^2^ = 0.039	*F*(1,195) = 2.392 *p* = 0.124 partial η^2^ = 0.012	*F*(1,193) = 0.020 *p* = 0.887 partial η^2^ < 0.001	*F*(1,193) = 0.749 *p* = 0.388 partial η^2^ = 0.004
IPL	*F*(1,195) = 1.506 *p* = 0.221 partial η^2^ = 0.008	*F*(1,195) = 2.502 *p* = 0.115 partial η^2^ = 0.013	*F*(1,193) = 1.817 *p* = 0.179 partial η^2^ = 0.009	*F*(1,193) = 4.136 *p* = 0.043 * partial η^2^ = 0.021
**SA**				
IFG	*F*(1,195) = 5.177 *p* = 0.024 * (R > L) partial η^2^ = 0.026	*F*(1,195) = 0.484 *p* = 0.487 partial η^2^ = 0.002	*F*(1,193) = 0.476 *p* = 0.491 partial η^2^ = 0.002	*F*(1,193) = 0.063 *p* = 0.801 partial η^2^ < 0.001
IPL	*F*(1,195) = 6.173 *p* = 0.014 * (L > R) partial η^2^ = 0.031	*F*(1,195) = 0.012 *p* = 0.912 partial η^2^ < 0.001	*F*(1,193) = 0.562 *p* = 0.455 partial η^2^ = 0.003	*F*(1,193) = 0.798 *p* = 0.373 partial η^2^ = 0.004

*p < 0.05; **p < 0.01; ***p < 0.001.

Key: GMV, gray matter volume; CT, cortical thickness; SA, surface area; IFG, inferior frontal gyrus; IPL, inferior parietal lobule; ANCOVA, Analysis of Covariance; R, right; L, left.

**TABLE 8:** Results for mixed ANCOVA models including hemisphere as within-subject factor for values of GMV, CT, and SA from two time points for the subsample of 154 participants.

	Basic ANCOVA model		Refined ANCOVA model	
	Hemisphere	Hemisphere × Time point × Language group	Hemisphere	Hemisphere × Time point × Language group
**GMV**				
IFG	*F*(1,151) = 0.175 *p* = 0.677 partial η^2^ = 0.001	*F*(1,151) = 0.043 *p* = 0.836 partial η^2^ < 0.001	*F*(1,147) = 0.655 *p* = 0.420 partial η^2^ = 0.004	*F*(1,147) = 0.030 *p* = 0.864 partial η^2^ < 0.001
IPL	*F*(1,151) = 11.116 *p* = 0.001 ** (L > R) partial η^2^ = 0.069	*F*(1,151) = 1.988 *p* = 0.161 partial η^2^ = 0.013	*F*(1,147) = 0.013 *p* = 0.908 partial η^2^ < 0.001	*F*(1,147) = 0.839 *p* = 0.361 partial η^2^ = 0.006
**CT**				
IFG	*F*(1,151) = 4.206 *p* = 0.042 * (L > R) partial η^2^ = 0.027	*F*(1,151) = 0.022 *p* = 0.881 partial η^2^ < 0.001	*F*(1,147) = 0.092 *p* = 0.762 partial η^2^ = 0.001	*F*(1,147) = 0.144 *p* = 0.705 partial η^2^ = 0.001
IPL	*F*(1,151) = 1.495 *p* = 0.223 partial η^2^ = 0.010	*F*(1,151) = 2.210 *p* = 0.139 partial η^2^ = 0.014	*F*(1,147) = 0.017 *p* = 0.896 partial η^2^ < 0.001	*F*(1,147) = 2.374 *p* = 0.126 partial η^2^ = 0.016
**SA**				
IFG	*F*(1,151) = 0.931 *p* = 0.336 partial η^2^ = 0.006	*F*(1,151) = 0.073 *p* = 0.788 partial η^2^ < 0.001	*F*(1,147) = 0.508 *p* = 0.477 partial η^2^ = 0.003	*F*(1,147) = 0.024 *p* = 0.878 partial η^2^ < 0.001
IPL	*F*(1,151) = 4.018 *p* = 0.047 * (L > R) partial η^2^ = 0.026	*F*(1,151) = 0.101 *p* = 0.751 partial η^2^ = 0.001	*F*(1,147) = 0.071 *p* = 0.790 partial η^2^ < 0.001	*F*(1,147) = 0.919 *p* = 0.339 partial η^2^ = 0.006

*p < 0.05; **p < 0.01.

Key: GMV, gray matter volume; CT, cortical thickness; SA, surface area; IFG, inferior frontal gyrus; IPL, inferior parietal lobule; ANCOVA, Analysis of Covariance; R, right; L, left.

#### 3.1.1. Analyses for GMV

##### 3.1.1.1. Total sample: basic ANCOVA models

In terms of group differences, there was significantly higher GMV in bilinguals compared to monolinguals in the IPL [left: *F*(1,195) = 9.966, *p* = 0.002; right: *F*(1,195) = 8.340, *p* = 0.004], but not in the IFG [left: *F*(1,195) = 0.007, *p* = 0.933; right: *F*(1,195) = 2.968, *p* = 0.087]. In all of the analyzed regions, younger adults displayed significantly higher GMV than older ones [IFG left: *F*(1,195) = 13.696, *p* < 0.001; IFG right: *F* (1,195) = 7.815, *p* = 0.006; IPL left: *F* (1,195) = 6.071, *p* = 0.015, IPL right: *F*(1,195) = 12.623, *p* < 0.001].

**FIGURE 2:**
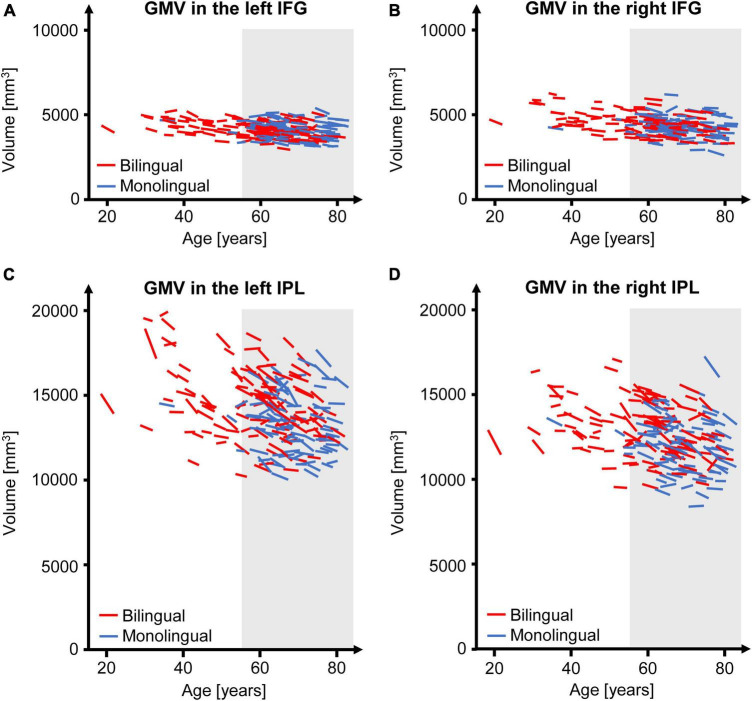
GMV change over time separately for each participant of the present study for the left **(A)** and right IFG **(B)** as well as for the left **(C)** and right IPL **(D)**. The gray underlay indicates participants ≥ 55 years at time point t1, who were included in the subsample. GMV, gray matter volume; IFG, inferior frontal gyrus; IPL, inferior parietal lobule.

**FIGURE 3:**
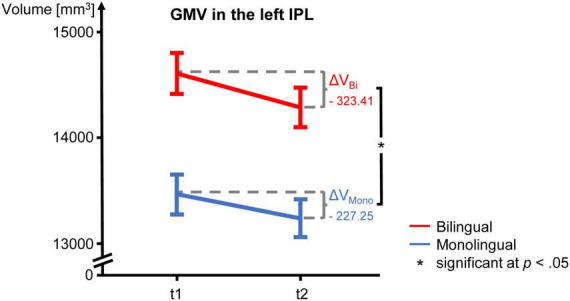
GMV change over time in mono- and bilinguals in the left IPL. For both language groups, mean GMV and standard error of the mean are depicted for t1 and t2 as well as ΔV, i.e., the mean absolute GMV difference between t1 and t2. The GMV decline over time is significantly steeper in bilinguals. GMV, gray matter volume; IPL, inferior parietal lobule; t1, first time point; t2, second time point.

The GMV change over time in the participants of the present study is depicted in Figure 2 for the analyzed regions. Regarding the interaction between language group and time point, GMV decline within the left IPL was significantly steeper in bilinguals when compared to monolinguals [*F*(1,195) = 4.211, *p* = 0.042] (Figure 3). In contrast, for the left and right IFG and the right IPL, bilinguals and monolinguals displayed a similar GMV change over time [IFG left: *F*(1,195) = 0.001, *p* = 0.976, IFG right: *F*(1,195) = 0.379, *p* = 0.539; IPL right: *F*(1,195) = 0.717, *p* = 0.398].

Adding hemisphere to the ANCOVA model revealed a significantly higher GMV in the left IPL compared to the right IPL [*F*(1,195) = 14.356, *p* < 0.001], while the left and right IFG displayed similar GMV values [*F*(1,195) = 2.144, *p* = 0.145]. There was no significant interaction effect for time point × language group × hemisphere neither for the IPL [*F*(1,195) = 2.490, *p* = 0.116] nor the IFG [*F*(1,195) = 0.265, *p* = 0.607]. For an overview of mean values and standard deviations for GMV at t1 and absolute GMV differences between t1 and t2 for the total sample, see Supplementary Tables 6, 7.

##### 3.1.1.2. Total sample: refined ANCOVA models

When including age, sex, education, and ICV as covariates, results for the bilateral IFG and the right IPL (see Table 4) corresponded to the ones emerging from the basic ANCOVA model. For the left IPL, no significant GMV difference between mono- and bilinguals could be found in these analyses [*F*(1,195) = 2.376, *p* = 0.125]. However, bilinguals still displayed a tendency for a steeper GMV decline within the left IPL when compared to monolinguals [*F*(1,195) = 3.475, *p* = 0.064].

Including hemisphere in the refined ANCOVA model yielded similar GMV values for the two hemispheres for both, the IPL and the IFG. Corresponding to basic ANCOVA analyses, no significant interaction effect for time point × language group × hemisphere was found, neither for the IPL, nor the IFG (see Table 7).

##### 3.1.1.3. Subsample analyses

Assessing participants ≥ 55 years only, we revealed similar results as obtained from the total sample for the bilateral IFG as well as the right IPL (see Tables 5, 6). For the left IPL, a higher GMV in bilinguals compared to monolinguals within the subsample was found for the basic ANCOVA model [*F*(1,195) = 8.703, *p* = 0.004], which remained a tendency when including age, sex, education, and ICV as covariates [*F*(1,195) = 3.517, *p* = 0.063]. However, there was no steeper GMV decline in bilinguals within the subsample for the basic [*F*(1,195) = 2.287, *p* = 0.133] nor the refined ANCOVA models [*F*(1,195) = 2.875, *p* = 0.092].

When including hemisphere as within-subject factor, results from both basic and refined ANCOVA model corresponded to the respective analyses of the total sample (see Table 8). For an overview of mean values and standard deviations for GMV at t1 and absolute GMV differences between t1 and t2 for the subsample, see Supplementary Tables 8, 9.

#### 3.1.2. Analyses for CT and SA

Results for the parallel analyses for CT and SA are presented in Tables 3–8. Regarding SA, bilinguals showed higher values than monolinguals in the bilateral IPL, but not IFG, within the basic analyses [total sample: IFG left: *F*(1,195) = 0.565, *p* = 0.453; IFG right: *F*(1,195) = 2.619, *p* = 0.107; IPL left: *F*(1,195) = 7.924, *p* = 0.005, IPL right: *F*(1,195) = 7.987, *p* = 0.005; subsample: IFG left: *F*(1,195) = 0.790, *p* = 0.375; IFG right: *F*(1,195) = 1.047, *p* = 0.308; IPL left: *F*(1,195) = 7.191, *p* = 0.008, IPL right: *F*(1,195) = 9.211, *p* = 0.003]. The higher SA in bilinguals within the right IPL was also stable in the refined ANCOVAs [total sample: *F*(1,195) = 7.493, *p* = 0.007; subsample: *F*(1,195) = 7.526, *p* = 0.007], thus largely mirroring results for GMV in terms of group differences. Over time, SA trajectories were similar for mono- and bilinguals in all analyses (see Tables 3–6).

For CT, similar values were found for mono- and bilinguals in all of the analyses (see Tables 3–6). Regarding trajectories over time, there was a significantly steeper CT decline in the left IPL in bilinguals compared to monolinguals [total sample: basic ANCOVA model: *F*(1,195) = 6.653, *p* = 0.011; refined ANCOVA model: *F*(1,195) = 4.700, *p* = 0.031]. When assessing the subsample, this steeper CT decline in bilinguals in the left IPL was only present on a trend level in the basic ANCOVA [*F*(1,195) = 3.244, *p* = 0.074], but significant for the refined ANCOVA [*F*(1,195) = 4.118, *p* = 0.044].

Additionally, in the refined total sample analyses including hemisphere as within-subject factor, a significant interaction effect between hemisphere, language group, and time point emerged for CT in the IPL [*F*(1,195) = 4.136, *p* = 0.043], but not the IFG [*F*(1,195) = 0.749, *p* = 0.388]. Thus, while the mean CT decline between t1 and t2 was steeper in bilinguals compared to monolinguals within the left IPL (monolinguals: −0.010 mm, bilinguals: −0.030 mm), it was similar for the two language groups within the right IPL (monolinguals: −0.027 mm; bilinguals: −0.028 mm). However, this interaction effect for CT in the left vs. right IPL was not significant in the basic ANCOVA models assessing the total sample [*F*(1,195) = 2.502, *p* = 0.115], nor in the subsample analyses [basic model: *F*(1,151) = 2.210, *p* = 0.139; refined model: IPL: *F*(1,147) = 2.374, *p* = 0.126].

### 3.2. Regression analyses

For regression analyses, similar results were revealed for analyses including and excluding ICV as predictor. In the following, results for analyses including ICV are reported (see also Supplementary Tables 10–21), while analyses excluding ICV are presented in Supplementary Tables 22–33.

#### 3.2.1. Analyses for GMV

When investigating the influence of AoA, LoP, and number of actively spoken languages on GMV in the bilateral IFG and IPL in bilinguals of the total sample, no significant effect of these predictors emerged for GMV at t1, nor t2, nor for GMV difference for any of the four ROIs (see Supplementary Tables 10, 11). The same results were found for bilinguals of the older subsample (see Supplementary Tables 16, 17).

#### 3.2.2. Analyses for CT

For CT in the left IFG, there was a tendency of later AoA predicting lower CT at t1 in bilinguals of the total sample (unstandardized coefficient *B* = −0.003; standard error = 0.002; *p* = 0.054). Additionally, higher LoP in this group predicted less CT decline between t1 and t2 for the left IFG (unstandardized coefficient *B* = 0.002; standard error = 0.001; *p* = 0.034) and there was a tendency for the very same effect within the left IPL (unstandardized coefficient *B* = 0.001; standard error = 0.001; *p* = 0.055). However, none of these effects showed significance when investigating bilinguals of the older subsample (see Supplementary Tables 18, 19).

For CT at t1 and t2 and CT differences in the right IFG, no effect of AoA, LoP and number of actively spoken languages was found, neither in bilinguals of the total sample (see Supplementary Table 12), nor of the subsample (see Supplementary Table 18). For the right IPL, a higher number of actively spoken languages showed a tendency of predicting higher CT at t2 within bilinguals of the subsample (unstandardized coefficient *B* = 0.035; standard error = 0.018; *p* = 0.059), while no effect of bilingual experience-based factors on CT at one of the two time points nor on CT differences was found within bilinguals of the total sample (see Supplementary Table 13).

#### 3.2.3. Analyses for SA

For the left IFG, later AoA was associated with higher SA at both time points in bilinguals of the total sample (for t1 only showing a tendency toward significance: unstandardized coefficient *B* = 3.118; standard error = 1.597; *p* = 0.054; t2: unstandardized coefficient *B* = 3.654; standard error = 1.607; *p* = 0.025) as well as in bilinguals of the older subsample (t1: unstandardized coefficient *B* = 4.152; standard error = 1.897; *p* = 0.032; t2: unstandardized coefficient *B* = 4.905; standard error = 1.861; *p* = 0.011). Additionally, later AoA predicted less SA decline between t1 and t2 within the left IFG (bilinguals of the total sample: unstandardized coefficient *B* = 0.221; standard error = 0.105; *p* = 0.037; bilinguals of the subsample: unstandardized coefficient *B* = 0.277; standard error = 0.118; *p* = 0.022).

For SA at t1 and t2 and SA differences in the right IFG as well as in the bilateral IPL, no effect of AoA, LoP and number of actively spoken languages was revealed, neither in bilinguals of the total sample (see Supplementary Tables 14, 15), nor of the subsample (see Supplementary Tables 20, 21).

## 4. Discussion

The present large-scale population-based study over two time points provides novel insights into the effects of long-term bilingualism on cortical brain structure. Five major results emerged: (1) For basic analyses of the total sample, there was a steeper GMV decline over time in bilinguals as compared to monolinguals in the left IPL, confirming the earlier cross-sectional observations (Heim et al., 2019) for the first time over two time points. However, this effect showed only a tendency toward significance when including age, sex, education, and ICV as covariates, and analyses of the older subsample yielded no significantly differing decline in mono- vs. bilinguals within the left IPL at all. (2) In both hemispheres, bilinguals showed a higher GMV in the IPL, but not the IFG, for basic analyses of the total sample, indicating that bilingualism might contribute to brain reserve especially in posterior brain regions. For refined analyses as well as subsample analyses, this effect was more stable for the right IPL. (3) With a steeper GMV decline in bilinguals as found in basic total sample analyses, GMV differences between monolinguals and bilinguals appear to diminish over time in the left IPL. In contrast, monolinguals and bilinguals showed a similar GMV change with aging in the right IPL, indicating that the bilingual brain reserve might be more persistent in the right IPL. (4) Analyses of CT and SA as the two factors of GMV revealed that, while SA appears to be the factor explaining the overall higher GMV in bilinguals in the bilateral IPL, CT explains more of the age-related changes in GMV than SA. For CT, there was also a steeper decline over time in bilinguals in the left IPL, corresponding to GMV trajectories in basic analyses of the total sample. In contrast to GMV, the differing CT trajectories for mono- vs. bilinguals within the left IPL were also stable for refined total sample analyses including age, sex, education, and ICV as covariates, as well as for refined subsample analyses. (5) For the left IFG, higher LoP was associated with less CT decline over time within bilinguals. Additionally, later AoA predicted higher SA in this brain region at both, t1 and t2, and was associated with less SA decline over time within bilinguals. Thus, even though monolinguals and bilinguals had shown similar GMV, CT, and SA within the left IFG when compared directly, bilingual experience-based factors such as AoA and LoP appear to modulate brain structure as well as trajectories of structural change over time within the left IFG.

### 4.1. The longitudinal effects of bilingualism on brain structure

Regarding GMV trajectories in mono- and bilinguals in the IPL, the present longitudinal results partially underpin those from cross-sectional studies. For the left IPL, a steeper GMV decline in bilinguals was found within the total sample for the basic ANCOVA, corresponding to results from Heim et al. (2019). This effect seemed to be especially attributable to aging-related CT changes. For the right IPL, however, trajectories of structural decline were similar for mono- and bilinguals in all of the analyses, which is in contrast to Abutalebi et al. (2015), who provided evidence for a steeper GMV decline in monolinguals in this region. While the left IPL is relevant for language processing, the right IPL has been associated with visuo-spatial attention reorientation as an aspect of executive functions (Numssen et al., 2021; for review, cf. Binkofski et al., 2016). Thus, the hypothesis proposed by Heim et al. (2019), that structural decline over time seems to be steeper in bilinguals in language-related areas, while bilingual brain reserve in the non-linguistic domain appears to be more persistent, is supported by the present data from two time points.

To provide explanations for the observed inter-hemispheric differences in GMV trajectories in mono- and bilinguals in the IPL in basic total sample analyses, two hypotheses will be discussed: First, a steeper GMV decline in bilinguals in the left IPL may represent increasing efficiency in bilinguals in language-related brain regions. A model that may provide a helpful framework for this hypothesis is the so-called “dynamic restructuring model” (DRM; Pliatsikas, 2020), which will be presented briefly. Second, a steeper decline in bilinguals may be expected in both, the left as well as the right IPL. However, due to age-related compensation strategies, such as increasing use of the right IPL with aging in bilinguals, structural decline in the right IPL may be attenuated in bilinguals.

According to the DRM (Pliatsikas, 2020), different cortical and subcortical adaptations in gray and white matter pertain to three different phases: (1) initial exposure, (2) consolidation, and (3) peak efficiency. The initial exposure to a new language results in an increase in cortical gray matter volume (GMV). During consolidation of the new skill, cortical GMV decreases again while subcortical and cerebellar GMV as well as white matter structural connectivity increase. The third stage, peak efficiency, implies further adaptations of cerebellar and subcortical GMV and white matter structural connectivity as a result of increasing proficiency and immersion in the additional language (DeLuca et al., 2019b; Pliatsikas, 2020).

The first hypothesis is predicated on the first two stages of structural plasticity in the bilingual brain, as proposed by the DRM: initial exposure and consolidation (Pliatsikas, 2020). As it is assumed that initial exposure to a new language induces GMV increases in cortical brain regions relevant for language and executive control, this may reflect a local generation of dendritic spines and new neural pathways during learning (Lövdén et al., 2013), possibly facilitated by an upregulation of neurotrophic factors such as noradrenaline (Robertson, 2013; Guzman-Velez and Tranel, 2015). Correspondingly, in the current study, bilinguals showed a higher GMV in the bilateral IPL compared to monolinguals (with this effect being more stable for the right IPL in the refined total sample analysis).

**FIGURE 4:**
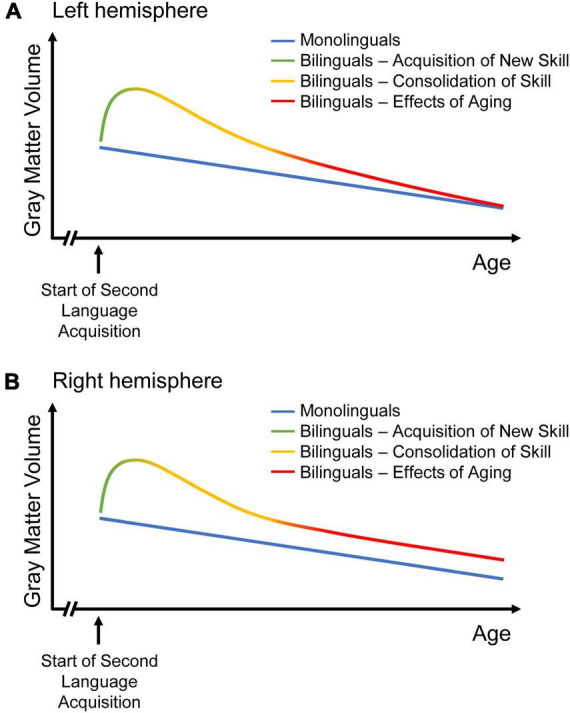
Two models of GMV change over the course of time in mono- and bilinguals. While GMV change in monolinguals is sketched as continuous GMV decline, three stages of dynamic structural change are depicted for bilinguals, as suggested in the “dynamic restructuring model” (DRM; Pliatsikas, 2020): Learning an additional language results in increasing GMV, followed by GMV decrease during the phase of consolidation. With aging, the decline in left-hemispheric language areas is steeper in bilinguals than in monolinguals **(A)**. Hence, the volume differences between monolinguals and bilinguals disappear over time, possibly reflecting an ongoing selection of most efficient neural circuits in bilinguals with continuous bilingual experience. Thus, bilingualism might result in constantly increasing efficiency with regards to language processing. In right-hemispheric regions related to domain-general control, the decrease in bilinguals attenuates until it matches monolingual decline **(B)**. Thus, bilingualism may provide a persistent brain reserve in the non-linguistic domain. Figures adapted from Heim et al. (2019).

With increasing proficiency and bilingual experience, a partial or complete return to baseline GMV takes place during the second phase, consolidation (Pliatsikas, 2020). This is supposed to reflect a selection of most efficient circuits, with non-efficient and therefore under-utilized spines being eliminated via pruning (Lövdén et al., 2013; Wenger et al., 2017). Thus, cortical GMV may decrease without loss of the novel skill (Wenger et al., 2017). In the present study, the steeper decline in bilinguals in the language-relevant left IPL might consequently reflect an ongoing selection of most efficient neural circuits due to a continuous bilingual experience. Nevertheless, one has to bear in mind that previous expansion-renormalization trajectories have been observed mostly in training studies over a course of weeks or months (Wenger et al., 2017; Pliatsikas, 2020), while the present data evaluate changes across a mean time interval ± SD of 3.6 ± 0.8 years after lifelong bilingual experience. Therefore, one might question whether the current findings could indeed correspond to continuously increasing efficiency in bilinguals or whether they might, contrarily, reflect an accelerated structural decline in aging bilinguals. However, if the steeper decline in bilinguals in the left IPL would actually reflect a continuous selection of most efficient neural circuits, corresponding to the stage of consolidation, then the DRM would predict parallel increases in cerebellar and subcortical GMV as well as greater structural connectivity in terms of white matter tracts in bilinguals (Pliatsikas, 2020). This might indicate a shift in weight from lexical acquisition (subserved by cortical regions) to language control (provided by subcortical and cerebellar structures) with increasing bilingual experience, the latter facilitated by efficient long-distance connectivity (Pliatsikas, 2020), which may be investigated in future studies. Figure 4 integrates the novel insights from the present analyses into the DRM.

The second hypothesis is based on the idea that bilingualism might modulate GMV trajectories not only in the left, but also in the right IPL. For the right IPL, which is relevant for executive functions such as visuo-spatial attention reorientation (Numssen et al., 2021; for review, cf. Binkofski et al., 2016), there was no steeper GMV decline in bilinguals as compared to monolinguals in the present study. Corresponding to the interpretation of GMV trajectories in the left IPL, a steeper GMV decline in bilinguals could have reflected increasing efficiency, while similar trajectories in mono- and bilinguals might indicate that bilingualism does not result in a continuous increase of efficiency in the domain of executive control. On the other hand, it is possible that not only language processing in the left IPL, but also the executive functions provided by the right IPL would become increasingly efficient in bilinguals over time. The increasing efficiency should be reflected in a steeper GMV decline in bilinguals due to pruning (Lövdén et al., 2013) and in increasing structural connectivity in bilinguals as proposed by the DRM (Pliatsikas, 2020). Simultaneously, however, GMV may increase in bilinguals due to an additional activation of the right IPL, for example in the context of aging-related compensation, to the extent that monolinguals and bilinguals show a similar GMV decline over time in the right IPL.

**FIGURE 5:**
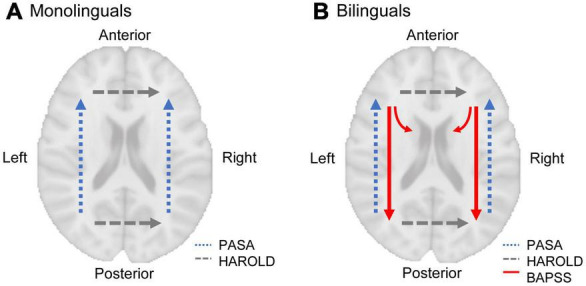
A model of shifts in task-induced neural activity in mono- and bilinguals in the course of aging (dashed lines) and bilingualism (solid lines). With aging, a “posterior-to-anterior-shift” (PASA; Davis et al., 2008) and a “hemispheric asymmetry reduction” (HAROLD; Cabeza, 2002) can be observed (in terms of language, which is usually processed in the left hemisphere, HAROLD predicts an increased recruitment of the right hemisphere with aging, as depicted here). In monolinguals, this leads to increasing prefrontal activation with aging **(A)**. In bilinguals, however, an “anterior-to-posterior and subcortical shift” (BAPSS; Grundy et al., 2017) occurs with increasing bilingual experience, which may counteract age-related changes predicted by PASA **(B)**. Therefore, BAPSS may represent a form of cognitive reserve in bilinguals. Additionally, the interaction of BAPSS and HAROLD may lead to increasing use of the right IPL with aging in bilinguals, but not monolinguals, possibly resulting in an attenuation of structural decline in bilinguals in the right IPL.

The interaction of three pre-existing models, that describe shifts in task-induced neural activity in the course of bilingualism and aging, may explain why monolinguals and bilinguals show a similar GMV decline over time in the right IPL (for a depiction of the models, see Figure 5). The models will be briefly presented in the following: (1) In bilinguals, a “bilingual anterior-to-posterior and subcortical shift” (BAPSS) can be observed with increasing second language experience (Grundy et al., 2017). This is interpreted as a shift from controlled (frontal) to automatic (posterior and subcortical) processing in bilinguals. As increasing activation of a certain brain region may result in an increase of GMV in the very same region (Lövdén et al., 2013), the higher GMV in bilinguals in the IPL found in the present study may reflect the more pronounced activation of posterior brain regions during cognitive processing in bilinguals. (2) With aging, however, a “posterior-to-anterior shift” (PASA) in neural activity has been described (Davis et al., 2008). This shift from automated to controlled processing has typically been interpreted as a compensatory mechanism to maintain cognitive functioning (Davis et al., 2008). In bilinguals, who seem to rely more on posterior (and subcortical) regions for processing, as described in BAPSS, anterior brain regions may remain available for age-related compensation as outlined in PASA (Davis et al., 2008) to a higher extent than in monolinguals (Grundy et al., 2017). Therefore, the bilingual brain reserve in the IPL, reflected by higher GMV, seems to be particularly beneficial (Heim et al., 2019), as it might support the capacity for compensation in frontal brain regions in bilinguals up to an older age than in monolinguals, thus possibly delaying age-related cognitive decline. (3) Additionally, the “hemispheric asymmetry reduction in older adults” (HAROLD) model states that in older adults, corresponding regions in both hemispheres are recruited for previously lateralized processes (Cabeza, 2002). This is suggested to reflect a compensatory process to maintain cognitive functioning despite age-related structural atrophy. In terms of language, which is usually processed in the left hemisphere, the HAROLD model would predict an increased recruitment of the right hemisphere with aging. An interaction of BAPSS and HAROLD may result in an attenuation of structural decline in bilinguals, but not monolinguals, in the right IPL: An increasing use of the right IPL with aging in bilinguals might compensate for GMV loss in the left IPL, as described in HAROLD (Cabeza, 2002). In turn, monolinguals, who seem to rely less on posterior brain regions than bilinguals (Grundy et al., 2017), might rather recruit frontal brain regions for compensation as described in PASA (Davis et al., 2008) than the right homologue of a posterior brain region. This could explain why bilinguals do not show a steeper decline than monolinguals in the right IPL, in contrast to GMV trajectories in the left IPL. Notably, while higher GMV in the IPL in bilinguals has been interpreted as a form of brain reserve, the above-described hypothesis might reflect a mechanism of brain maintenance in bilinguals, possibly resulting in a reduced structural decline over time in bilinguals in the right IPL.

The two hypotheses proposed to explain the inter-hemispheric differences in structural decline in the bilingual brain do not mutually exclude each other. Instead, they touch upon complementary aspects of the same topic, since the first hypothesis focuses on the steeper decline in the left IPL in bilinguals and the second on the (presumably) attenuated decline in the right IPL. Future research is warranted to examine whether these hypotheses prove true. Additionally, one has to bear in mind that GMV trajectories in the left and right IPL in mono- and bilinguals did not differ significantly from each other when compared directly. However, as the interaction between hemisphere, language group, and time point was significant for CT for the refined total sample analyses in this brain region, the results from CT analyses underline the idea of inter-hemispheric differences regarding structural change in the bilingual brain during aging. Furthermore, there was only a tendency of a steeper GMV decline in bilinguals within the left IPL when age, sex, education, and ICV were included into the ANCOVA model as covariates, and analyses of the subsample including only participants ≥ 55 years revealed no significantly differing decline in mono- vs. bilinguals at all. For CT, however, the decline remained significantly steeper in bilinguals not only when including the additional covariates in the total sample analyses, but also for the refined analyses of participants ≥ 55 years. Thus, SA appears to be the factor contributing to higher GMV in bilinguals in cortical brain regions corresponding to previous studies (Li et al., 2017), but CT might be the one mediating the effects of aging on GMV.

In contrast to cross-sectional results, which indicated higher GMV in bilinguals in the left and right IFG and a steeper GMV decline in bilinguals in the left IFG (Heim et al., 2019), group differences between mono- and bilinguals within the bilateral IFG are missing in the present data. On one hand, the previous cross-sectional study might have overestimated the effect of bilingualism on GMV in the IFG. On the other hand, the smaller number of participants in the present study compared to the cross-sectional sample of Heim et al. (2019), resulting in a reduced statistical power, might explain the discrepancy. However, regression analyses revealed that AoA and LoP, factors modulating the bilingual experience, seem to have an impact on structural parameters and their trajectories over time within the left IFG in bilinguals of the present sample. Previous studies also showed an effect of these experience-based factors on bilingual brain structure in the left IFG, albeit partially contradictory to the present results: for AoA, for example, Klein et al. (2014) found that later AoA was associated with higher CT in the left and less CT in the right IFG, while there was a tendency of later AoA predicting less CT within the left IFG for the current study. Additionally, for bilinguals of the present sample, later AoA was associated with higher SA in this brain region at both, t1 and t2, and predicted less SA decline over time. For LoP, the present study revealed less CT decline over time with higher LoP. Thus, viewing bilingualism as a continuous spectrum of experiences modulated by factors such as AoA and LoP may reveal effects that do not become evident when performing only dichotomous group comparisons between mono- and bilinguals (see also DeLuca et al., 2019a,^2020^).

For the bilateral IPL, AoA, LoP and number of actively spoken languages showed no significant effects on brain structure within bilinguals of the present sample. Previously, earlier AoA and higher LoP have been associated with higher gray matter density in the left (and for AoA, also in the right) IPL (Mechelli et al., 2004). However, when investigating GMV in the bilateral IPL in older bilinguals (mean age ± SD: 63.2 ± 5.86), Abutalebi et al. (2015) observed no effect of AoA on GMV, corresponding to the current findings. Thus, it is possible that the importance of AoA for the modulation of adaptations to bilingualism may diminish in the case of lifelong second language use (Abutalebi et al., 2015).

Altogether, a similar pattern emerges from cross-sectional and longitudinal observations when taking results for both, IFG and IPL, into account: Bilingualism appears to add brain reserve, expressed by higher GMV, especially to posterior brain regions (Heim et al., 2019). Additionally, there seems to be a steeper structural decline in bilinguals in the left, but not in the right hemisphere (Abutalebi et al., 2015; Heim et al., 2019). The latter finding, possibly reflecting a reduced structural decline in bilinguals in the right IPL, could correspond to a form of brain maintenance in bilinguals. Yet, further longitudinal studies are necessary to explore the impact of bilingualism on structural change in the human brain over time in greater detail.

### 4.2. Limitations and future directions

There are some limitations of the current study that should be mentioned. First, because the present sample included mainly native German speakers, the LEAP-Q was chosen to evaluate participants’ second language status, as there is a German version available. The use of an alternative instrument would have most likely made it necessary not only to translate, but also validate said instrument. However, the LEAP-Q as a self-assessment questionnaire does not include objective evaluation of second language abilities, which may be considered a disadvantage. Nonetheless, the LEAP-Q has shown external validity when compared to objective measures of language proficiency (Marian et al., 2007) and may therefore be regarded a reliable tool for the evaluation of language abilities in the context of the present study.

Second, the present results are based on structural MRI data from two time points only. Thus, further studies encompassing more than two time points are necessary. Additionally, future longitudinal studies could take more than one parameter into account – for example, analyses of GMV could be combined with analyses of functional and structural connectivity or with neurocognitive data –, thus eventually providing an integrated view on longitudinal changes in the brains of long-term bilinguals across multiple modalities.

Third, bilinguals were mostly native German speakers with variable second language background, and while Germanic, Italic and Balto-Slavic languages could be included in the present study (see Table 2), it is unclear whether the current results would be generalizable for any combination of languages. In a previous cross-sectional study, Abutalebi et al. (2015) investigated GMV in the bilateral IPL in 30 monolinguals compared to 16 Cantonese-English and 14 Cantonese-Mandarin bilinguals. They found higher GMV in bilinguals, corresponding to the present results, but evidence for a steeper GMV decline in monolinguals in the right IPL, in contrast to the present finding of a steeper GMV decline over time in bilinguals in the left IPL. When investigating the influence of linguistic distance on GMV, there was a trend toward significance for the association between second language naming performance and GMV in the left IPL only for Cantonese-Mandarin bilinguals, which was interpreted as possible evidence for increased control demands in bilinguals who speak typologically close languages (Abutalebi et al., 2015). However, for the right IPL, a significant correlation between language exposure and GMV was revealed for both Cantonese-English and Cantonese-Mandarin bilinguals (Abutalebi et al., 2015). Thus, one might assume some consistencies in structural brain adaptations to bilingualism (cf. Danylkiv and Krafnick, 2020), but further large-scale longitudinal studies are necessary to test whether the present finding of a putatively steeper structural decline in bilinguals in the left IPL is generalizable across varying language combinations.

## 5. Conclusion

To the best of our knowledge, the present study is the first to investigate age-related GMV changes in bilinguals as compared to monolinguals in a longitudinal approach within a large sample. Importantly, the cross-sectional observations of a steeper GMV decline over time in bilinguals when compared to monolinguals were confirmed over two time points for the left IPL. Additionally, as there was a higher GMV in bilinguals in the IPL, but not the IFG, our results indicate that bilingualism might contribute to brain reserve especially in posterior brain regions. With the steeper GMV decline in bilinguals, which appears to be mediated by CT rather than SA, the volume differences between monolinguals and bilinguals might diminish over time in the left IPL. However, there appears to be a higher persistence of brain reserve in bilinguals in the right IPL. Furthermore, experience-based factors such as AoA and LoP appear to modulate brain structure as well as trajectories of structural change over time in bilinguals within the left IFG. Altogether, the importance of longitudinal studies when investigating the effects of bilingualism on structural features of the human brain becomes evident.

## Data availability statement

The raw data supporting the conclusions of this article will be made available by the authors upon request, without undue reservation.

## Ethics statement

The studies involving human participants were reviewed and approved by the local Ethics Committee of the University of Essen, Germany. The participants provided their written informed consent to participate in this study.

## Author contributions

KP: methodology, formal analysis, visualization, writing–original draft, and writing–reviewing and editing. JS, CJ, and NB: methodology, investigation, data curation, and writing–reviewing and editing. SC: conceptualization, resources, project administration, funding acquisition, and writing–reviewing and editing. SH: supervision, conceptualization, methodology, project administration, and writing–reviewing and editing. All authors contributed to the article and approved the submitted version.

## Footnotes

1 In the present study, the terms “bilingualism” and “bilinguals” refer to situations where people speak two or *more than* two languages, since many individuals learn a third or fourth language over time (cf. Li et al., 2014).

2
https://www.ibm.com/docs/en/spss-statistics/27.0.0
