# Supplementary material for: The influence of bilingualism on gray matter volume in the course of aging: a longitudinal study
Source: Front Aging Neurosci. 2023 Jul 20;15:1193283. doi: 10.3389/fnagi.2023.1193283 (PMC10400456; doi:10.3389/fnagi.2023.1193283)
Supplement: Supplementary file 1 [file Data_Sheet_1.pdf]

Supplementary Material

The influence of bilingualism on gray matter volume in the course of aging: A longitudinal study

Katharina Peitz*, Johanna Stumme, Christiane Jockwitz, Nora Bittner, Svenja Caspers & Stefan Heim

*** Correspondence:** Katharina Peitz: k.peitz@fz-juelich.de, katharina.peitz@hhu.de

1 Supplementary Methods

1.1 Cross-sectional vs. longitudinal sample

Both samples, the one from the cross-sectional study that reported a steeper GMV decline over time in bilinguals (Heim et al., 2019) as well as the current longitudinal sample, were drawn from the 1000BRAINS cohort. While 200 participants constituted the longitudinal sample with two complete data points each (Table 1), 399 individuals with one complete data point each were included in the cross-sectional study (Heim et al., 2019). As data acquisition for 1000BRAINS continued after the cross-sectional analysis, the longitudinal cohort is not just a subset of the cross-sectional sample, but also includes participants that were not part of the cross-sectional study. There were 113 participants (63 monolinguals, 50 bilinguals) who were part of both cohorts. In order to be able to relate the findings from the present longitudinal to the former cross-sectional analysis, the comparability of the two samples as well as of the monolinguals and bilinguals of each sample was assessed using the chi-square test for categorical variables, independent-samples t-tests for interval-scaled variables, or Welch's t-test for interval-scaled variables with unequal variances. The data were comparable to the largest extent except for education (in years; assessment of the educational level was based on the International Standard Classification of Education; UNESCO Institute for Statistics, 2012), which was significantly higher in the full longitudinal sample ($t[597] = -3.127$, $p = 0.002$) and also in the subset of monolinguals of the longitudinal sample ($t[146.168] = -1.992$, $p = 0.048$) when compared to their cross-sectional counterparts (Table S2).

2 Supplementary Tables

Supplementary Table 1. Study sample (n = 200): demographic characteristics of the age groups (younger/older participants, divided at the age median)

	Monolinguals (n = 87)		Bilinguals (n = 113)	
	Younger (< 62.8 y, n = 28)	Older (> 62.8 y, n = 59)	Younger (< 62.8 y, n = 72)	Older (> 62.8 y, n = 41)
Gender				
% Female	60.7	37.3	41.7	41.5
% Male	39.3	62.7	58.3	58.5
Age at t1 [years]				
Mean (SD)	58.2 (5.6)	70.2 (5.0)	49.8 (10.2)	69.0 (4.2)
Minimum	33.7	62.9	18.5	63.1
Maximum	62.6	79.4	62.3	78.4
Education level at t1 (SD)	6.2 (1.8)	5.9 (1.8)	7.4 (1.7)	8.0 (1.8)
Time interval between t1 and t2 (SD) [years]	3.9 (0.9)	3.7 (0.7)	3.3 (0.8)	3.5 (0.8)

Group characteristics for monolinguals and bilinguals for younger (below median) and older (above median) participants of the total sample. Key: t1, first time point; t2, second time point; SD, standard deviation; y, years.

Supplementary Table 2. Results for statistical tests comparing the current longitudinal sample with the cross-sectional sample of Heim et al. (2019)

	Total (CS: n = 399; LS: n = 200)		Monolinguals (CS: n = 224; LS: n = 87)		Bilinguals (CS: n = 175; LS: n = 113)	
Age[1]	$t[597] = 1.787$	$p = 0.074$	$t[309] = 0.093$	$p = 0.926$	$t[286] = 0.791$	$p = 0.430$
Sex[2]	$\chi^2[1] = 3.311$	$p = 0.069$	$\chi^2[1] = 1.545$	$p = 0.214$	$\chi^2[1] = 1.347$	$p = 0.246$
Education[1, 3]	$t[597] = -3.127$	$p = 0.002$ **	$t[146.168] = -1.992$	$p = 0.048$ *	$t[286] = -0.809$	$p = 0.419$
Number of forgotten languages[1]	$t[597] = -1.015$	$p = 0.311$	$t[309] = -0.840$	$p = 0.402$	$t[286] = -0.385$	$p = 0.701$
Number of actively used languages[1]	–	–	–	–	$t[286] = 0.406$	$p = 0.685$
AoA[1]	–	–	–	–	$t[286] = -0.679$	$p = 0.498$
LoP[1]	–	–	–	–	$t[286] = 0.060$	$p = 0.952$

For the longitudinal sample, data from the first time point were submitted to the analyses. For three variables (number of actively used languages, AoA, and LoP), comparability of the two samples was only assessed for bilinguals.
*$p < 0.05$; **$p < 0.01$; ***$p < 0.001$.
[1] Assessment via independent-samples t-test; [2] assessment via χ^2 test; [3] for monolinguals, assessment via Welch's t-test due to unequal variances.
Key: CS, cross-sectional sample, LS, longitudinal sample, AoA, age of acquisition, LoP, level of proficiency.

Supplementary Table 3. Results for refined mixed ANCOVA models for values of CT and SA from two time points for 200 participants, including age, sex, education, and time interval as covariates

	Left hemisphere		Right hemisphere	
	Language group	Time point × Language group	Language group	Time point × Language group
GMV				
IFG	$F[1, 194] = 0.586$<br>$p = 0.445$<br>partial $\eta^2 = 0.003$	$F[1, 194] = 0.049$<br>$p = 0.824$<br>partial $\eta^2 < 0.001$	$F[1, 194] = 0.108$<br>$p = 0.743$<br>partial $\eta^2 = 0.001$	$F[1, 194] = 0.564$<br>$p = 0.454$<br>partial $\eta^2 = 0.003$
IPL	$F[1, 194] = 2.767$<br>$p = 0.098$<br>partial $\eta^2 = 0.014$	$F[1, 194] = 3.772$<br>$p = 0.054$<br>partial $\eta^2 = 0.019$	$F[1, 194] = 4.262$<br>$p = 0.040$ *<br>partial $\eta^2 = 0.021$	$F[1, 194] = 0.452$<br>$p = 0.502$<br>partial $\eta^2 = 0.002$
CT				
IFG	$F[1, 194] = 0.002$<br>$p = 0.962$<br>partial $\eta^2 < 0.001$	$F[1, 194] = 0.284$<br>$p = 0.595$<br>partial $\eta^2 = 0.001$	$F[1, 194] = 1.133$<br>$p = 0.289$<br>partial $\eta^2 = 0.006$	$F[1, 194] = 0.246$<br>$p = 0.620$<br>partial $\eta^2 = 0.001$
IPL	$F[1, 194] = 0.475$<br>$p = 0.491$<br>partial $\eta^2 = 0.002$	$F[1, 194] = 4.980$<br>$p = 0.027$ *<br>partial $\eta^2 = 0.025$	$F[1, 194] = 1.036$<br>$p = 0.310$<br>partial $\eta^2 = 0.005$	$F[1, 194] = 0.026$<br>$p = 0.872$<br>partial $\eta^2 < 0.001$
SA				
IFG	$F[1, 194] = 0.999$<br>$p = 0.319$<br>partial $\eta^2 = 0.005$	$F[1, 194] = 0.551$<br>$p = 0.459$<br>partial $\eta^2 = 0.003$	$F[1, 194] = 0.511$<br>$p = 0.475$<br>partial $\eta^2 = 0.003$	$F[1, 194] = 0.127$<br>$p = 0.722$<br>partial $\eta^2 = 0.001$
IPL	$F[1, 194] = 1.729$<br>$p = 0.190$<br>partial $\eta^2 = 0.009$	$F[1, 194] = 0.257$<br>$p = 0.613$<br>partial $\eta^2 = 0.001$	$F[1, 194] = 6.939$<br>$p = 0.009$ **<br>partial $\eta^2 = 0.035$	$F[1, 194] = 0.158$<br>$p = 0.691$<br>partial $\eta^2 = 0.001$

GMV analyses are reported as well for the sake of comparison.
*$p < 0.05$; **$p < 0.01$; ***$p < 0.001$.
Key: GMV, gray matter volume; CT, cortical thickness; SA, surface area; IFG, inferior frontal gyrus; IPL, inferior parietal lobule; ANCOVA, Analysis of Covariance.

Supplementary Table 4. Results for refined mixed ANCOVA models for values of CT and SA from two time points for the subsample of 154 participants, including age, sex, education, and time interval as covariates

	Left hemisphere		Right hemisphere	
	Language group	Time point × Language group	Language group	Time point × Language group
GMV				
IFG	$F[1, 148] = 0.391$<br>$p = 0.533$<br>partial $\eta^2 = 0.003$	$F[1, 148] = 0.118$<br>$p = 0.732$<br>partial $\eta^2 = 0.001$	$F[1, 148] = 0.189$<br>$p = 0.664$<br>partial $\eta^2 = 0.001$	$F[1, 148] = 0.444$<br>$p = 0.506$<br>partial $\eta^2 = 0.003$
IPL	$F[1, 148] = 3.996$<br>$p = 0.047$ *<br>partial $\eta^2 = 0.026$	$F[1, 148] = 3.300$<br>$p = 0.071$<br>partial $\eta^2 = 0.022$	$F[1, 148] = 5.230$<br>$p = 0.024$ *<br>partial $\eta^2 = 0.034$	$F[1, 148] = 1.244$<br>$p = 0.266$<br>partial $\eta^2 = 0.008$
CT				
IFG	$F[1, 148] = 0.051$<br>$p = 0.822$<br>partial $\eta^2 < 0.001$	$F[1, 148] = 0.119$<br>$p = 0.731$<br>partial $\eta^2 = 0.001$	$F[1, 148] = 1.489$<br>$p = 0.224$<br>partial $\eta^2 = 0.010$	$F[1, 148] = 0.022$<br>$p = 0.882$<br>partial $\eta^2 < 0.001$
IPL	$F[1, 148] = 0.498$<br>$p = 0.482$<br>partial $\eta^2 = 0.003$	$F[1, 148] = 4.562$<br>$p = 0.034$ *<br>partial $\eta^2 = 0.030$	$F[1, 148] = 0.461$<br>$p = 0.498$<br>partial $\eta^2 = 0.003$	$F[1, 148] = 0.179$<br>$p = 0.673$<br>partial $\eta^2 = 0.001$
SA				
IFG	$F[1, 148] = 0.881$<br>$p = 0.349$<br>partial $\eta^2 = 0.006$	$F[1, 148] = 0.390$<br>$p = 0.533$<br>partial $\eta^2 = 0.003$	$F[1, 148] = 0.920$<br>$p = 0.339$<br>partial $\eta^2 = 0.006$	$F[1, 148] = 0.461$<br>$p = 0.498$<br>partial $\eta^2 = 0.003$
IPL	$F[1, 148] = 2.487$<br>$p = 0.117$<br>partial $\eta^2 = 0.017$	$F[1, 148] = 0.091$<br>$p = 0.764$<br>partial $\eta^2 = 0.001$	$F[1, 148] = 7.196$<br>$p = 0.008$ **<br>partial $\eta^2 = 0.046$	$F[1, 148] = 0.387$<br>$p = 0.535$<br>partial $\eta^2 = 0.003$

GMV analyses are reported as well for the sake of comparison.
*$p < 0.05$; **$p < 0.01$; ***$p < 0.001$.
Key: GMV, gray matter volume; CT, cortical thickness; SA, surface area; IFG, inferior frontal gyrus; IPL, inferior parietal lobule; ANCOVA, Analysis of Covariance.

Supplementary Table 5. Results for refined mixed ANCOVA models including hemisphere as within-subject factor and age, sex, education, and time interval as covariates for values of CT and SA from two time points for both total sample and older subsample

	Total sample (n = 200)		Older subsample (n = 154)	
	Hemisphere	Hemisphere × Time point × Language group	Hemisphere	Hemisphere × Time point × Language group
GMV				
IFG	$F[1, 194] = 6.030$<br>$p = 0.015$ * (R > L)<br>partial $\eta^2 = 0.030$	$F[1, 194] = 0.148$<br>$p = 0.701$<br>partial $\eta^2 = 0.001$	$F[1, 148] = 0.448$<br>$p = 0.504$<br>partial $\eta^2 = 0.003$	$F[1, 148] = 0.041$<br>$p = 0.841$<br>partial $\eta^2 < 0.001$
IPL	$F[1, 194] = 8.863$<br>$p = 0.003$ ** (L > R)<br>partial $\eta^2 = 0.044$	$F[1, 194] = 2.529$<br>$p = 0.113$<br>partial $\eta^2 = 0.013$	$F[1, 148] = 1.059$<br>$p = 0.305$<br>partial $\eta^2 = 0.007$	$F[1, 148] = 1.126$<br>$p = 0.290$<br>partial $\eta^2 = 0.008$
CT				
IFG	$F[1, 194] = 5.338$<br>$p = 0.022$ * (L > R)<br>partial $\eta^2 = 0.027$	$F[1, 194] = 0.842$<br>$p = 0.360$<br>partial $\eta^2 = 0.004$	$F[1, 148] = 0.196$<br>$p = 0.659$<br>partial $\eta^2 = 0.001$	$F[1, 148] = 0.199$<br>$p = 0.656$<br>partial $\eta^2 = 0.001$
IPL	$F[1, 194] = 0.131$<br>$p = 0.718$<br>partial $\eta^2 = 0.001$	$F[1, 194] = 4.552$<br>$p = 0.034$ *<br>partial $\eta^2 = 0.023$	$F[1, 148] = 0.464$<br>$p = 0.497$<br>partial $\eta^2 = 0.003$	$F[1, 148] = 2.796$<br>$p = 0.097$<br>partial $\eta^2 = 0.019$
SA				
IFG	$F[1, 194] = 8.498$<br>$p = 0.004$ ** (R > L)<br>partial $\eta^2 = 0.042$	$F[1, 194] = 0.081$<br>$p = 0.776$<br>partial $\eta^2 < 0.001$	$F[1, 148] = 0.712$<br>$p = 0.400$<br>partial $\eta^2 = 0.005$	$F[1, 148] = 0.022$<br>$p = 0.883$<br>partial $\eta^2 < 0.001$
IPL	$F[1, 194] = 3.313$<br>$p = 0.070$<br>partial $\eta^2 = 0.017$	$F[1, 194] = 0.826$<br>$p = 0.365$<br>partial $\eta^2 = 0.004$	$F[1, 148] = 0.535$<br>$p = 0.466$<br>partial $\eta^2 = 0.004$	$F[1, 148] = 0.975$<br>$p = 0.325$<br>partial $\eta^2 = 0.007$

GMV analyses are reported as well for the sake of comparison.
*$p < 0.05$; **$p < 0.01$; ***$p < 0.001$.
Key: GMV, gray matter volume; CT, cortical thickness; SA, surface area; IFG, inferior frontal gyrus; IPL, inferior parietal lobule; ANCOVA, Analysis of Covariance; R, right; L, left.

Supplementary Table 6. GMV in the left hemisphere for 200 participants: mean values and standard deviations

	Monolinguals		Bilinguals	
	Mean value at t1 (SD)	Mean absolute difference (SD)	Mean value at t1 (SD)	Mean absolute difference (SD)
GMV [mm^3]				
IFG	4096.68 (549.96)	-81.80 (112.50)	4176.68 (484.43)	-76.75 (121.18)
IPL	13466.63 (1763.62)	-227.25 (339.41)	14611.17 (2094.40)	-323.41 (383.56)
CT [mm]				
IFG	2.639 (0.151)	-0.006 (0.080)	2.702 (0.141)	-0.019 (0.061)
IPL	2.546 (0.120)	-0.010 (0.057)	2.614 (0.128)	-0.030 (0.056)
SA [mm^2]				
IFG	1288.61 (168.21)	-21.89 (41.22)	1276.35 (138.59)	-13.41 (27.46)
IPL	4717.98 (629.51)	-44.45 (79.56)	4970.69 (685.26)	-35.10 (62.87)

Mean values and standard deviations (SD) for GMV at t1, absolute GMV differences between t1 and t2 and the GMV percentage change between t1 and t2 for the left hemisphere in monolinguals and bilinguals for the total sample. Values for CT and SA are reported to provide a broader picture.

Supplementary Table 7. GMV in the right hemisphere for 200 participants: mean values and standard deviations

	Monolinguals		Bilinguals	
	Mean value at t1 (SD)	Mean absolute difference (SD)	Mean value at t1 (SD)	Mean absolute difference (SD)
GMV [mm^3]				
IFG	4287.76 (594.53)	-78.79 (113.00)	4512.28 (628.96)	-61.25 (95.87)
IPL	12127.51 (1582.03)	-216.62 (280.07)	13026.85 (1631.12)	-221.05 (317.84)
CT [mm]				
IFG	2.593 (0.134)	-0.008 (0.073)	2.627 (0.128)	-0.005 (0.054)
IPL	2.560 (0.123)	-0.027 (0.066)	2.599 (0.134)	-0.028 (0.062)
SA [mm^2]				
IFG	1405.20 (192.22)	-22.10 (44.11)	1453.81 (191.56)	-16.11 (32.28)
IPL	4252.77 (551.32)	-20.92 (99.31)	4487.22 (538.63)	-20.94 (87.70)

Mean values and standard deviations (SD) for GMV at t1, absolute GMV differences between t1 and t2 and the GMV percentage change between t1 and t2 for the right hemisphere in monolinguals and bilinguals for the total sample. Values for CT and SA are reported to provide a broader picture.

Supplementary Table 8. GMV in the left hemisphere for 154 participants: mean values and standard deviations

	Monolinguals		Bilinguals	
	Mean value at t1 (SD)	Mean absolute difference (SD)	Mean value at t1 (SD)	Mean absolute difference (SD)
GMV [mm^3]				
IFG	4093.90 (550.01)	-85.39 (113.53)	4035.93 (457.99)	-67.58 (126.49)
IPL	13459.82 (1789.41)	-234.66 (340.24)	14306.77 (1893.03)	-304.51 (375.96)
CT [mm]				
IFG	2.637 (0.147)	-0.008 (0.080)	2.657 (0.132)	-0.010 (0.060)
IPL	2.543 (0.118)	-0.010 (0.057)	2.565 (0.107)	-0.024 (0.054)
SA [mm^2]				
IFG	1287.58 (169.22)	-22.30 (41.18)	1258.44 (144.17)	-17.28 (29.22)
IPL	4718.52 (627.18)	-45.57 (80.85)	4972.90 (666.86)	-38.85 (64.38)

Mean values and standard deviations (SD) for GMV at t1, absolute GMV differences between t1 and t2 and the GMV percentage change between t1 and t2 for the left hemisphere in monolinguals and bilinguals for the subsample of participants ≥ 55 years. Values for CT and SA are reported to provide a broader picture.

Supplementary Table 9. GMV in the right hemisphere for 154 participants: mean values and standard deviations

	Monolinguals		Bilinguals	
	Mean value at t1 (SD)	Mean absolute difference (SD)	Mean value at t1 (SD)	Mean absolute difference (SD)
GMV [mm^3]				
IFG	4284.29 (605.92)	-80.10 (114.52)	4339.55 (551.82)	-64.28 (95.50)
IPL	12109.89 (1607.57)	-217.22 (283.41)	12802.93 (1579.15)	-215.45 (293.35)
CT [mm]				
IFG	2.590 (0.135)	-0.006 (0.074)	2.589 (0.112)	-0.004 (0.062)
IPL	2.555 (0.118)	-0.027 (0.067)	2.555 (0.117)	-0.028 (0.065)
SA [mm^2]				
IFG	1404.34 (195.85)	-23.81 (44.17)	1423.65 (172.84)	-17.90 (37.89)
IPL	4253.42 (561.10)	-19.70 (101.35)	4509.07 (556.04)	-18.89 (105.63)

Mean values and standard deviations (SD) for GMV at t1, absolute GMV differences between t1 and t2 and the GMV percentage change between t1 and t2 for the right hemisphere in monolinguals and bilinguals for the subsample of participants $\geq$ 55 years. Values for CT and SA are reported to provide a broader picture.

Supplementary Table 10. Results for regression analyses for GMV in the IFG as dependent variable for bilinguals of the total sample (n = 113)

	Left IFG			Right IFG		
	GMV at t1	GMV at t2	GMV difference	GMV at t1	GMV at t2	GMV difference
General model statistics	$R^2 = 0.374$ F[7, 105] = 8.972 p < 0.001***	$R^2 = 0.364$ F[7, 105] = 8.586 p < 0.001***	$R^2 = 0.124$ F[8, 104] = 1.835 p = 0.079	$R^2 = 0.470$ F[7, 105] = 13.279 p < 0.001***	$R^2 = 0.467$ F[7, 105] = 13.165 p < 0.001***	$R^2 = 0.136$ F[8, 104] = 2.049 p = 0.048 *
**Predictors**	**B (SE)**	**B (SE)**	**B (SE)**	**B (SE)**	**B (SE)**	**B (SE)**
AoA	3.297 (5.453)	6.083 (5.306)	0.764 (0.483)	2.915 (6.518)	1.611 (6.404)	-0.006 (0.377)
LoP	-2.811 (14.863)	1.954 (14.495)	1.612 (1.303)	4.875 (17.767)	9.578 (17.494)	1.221 (1.018)
NoL	2,375 (55.641)	-19.369 (54.584)	-5.905 (4.913)	-47.944 (66.513)	-43.870 (65.880)	2.021 (3.840)
Age	-16.721 (3.377) ***	-14.089 (3.268) ***	0.811 (0.294) **	-22.144 (4.037) ***	-20.540 (3.945) ***	0.305 (0.230)
Sex (males = 0, females = 1)	-71.032 (95.990)	-4.939 (94.409)	15.451 (8.614)	-101.490 (114.747)	-39.764 (113.946)	16.792 (6.732) *
Education	8.254 (24.291)	17.553 (23.698)	1.597 (2.113)	9.888 (29.038)	3.752 (28.602)	-1.750 (1.652)
ICV	1.464 (0.348) ***	1.595 (0.345) ***	0.022 (0.031))	2.197 (0.416) ***	2.272 (0.416) ***	0.011 (0.024)
Time interval	-	-	-2.902 (4.397)	-	-	-5.095 (3.437)
Constant	2879.685 (641.863) ***	2361.865 (648.612) ***	-124.316 (59.745) *	2446.008 (767.284) **	2236.992 (782.836) **	-42.265 (46.695)

*$p < 0.05$; **$p < 0.01$; ***$p < 0.001$.
Key: GMV, gray matter volume; IFG, inferior frontal gyrus; AoA, age of acquisition; LoP, level of proficiency; NoL, number of actively spoken languages; ICV, intracranial volume; B, unstandardized coefficient B; SE, standard error.

Supplementary Table 11. Results for regression analyses for GMV in the IPL as dependent variable for bilinguals of the total sample (n = 113)

	Left IPL			Right IPL		
	GMV at t1	GMV at t2	GMV difference	GMV at t1	GMV at t2	GMV difference
General model statistics	$R^2 = 0.508$<br>F[7, 105] = 15.474<br>p < 0.001***	$R^2 = 0.498$<br>F[7, 105] = 14.896<br>p < 0.001***	$R^2 = 0.074$<br>F[8, 104] = 1.045<br>p = 0.408	$R^2 = 0.482$<br>F[7, 105] = 13.954<br>p < 0.001***	$R^2 = 0.457$<br>F[7, 105] = 12.605<br>p < 0.001***	$R^2 = 0.112$<br>F[8, 104] = 1.638<br>p = 0.123
**Predictors**	**B (SE)**	**B (SE)**	**B (SE)**	**B (SE)**	**B (SE)**	**B (SE)**
AoA	-11.211 (20.910)	-11.631 (20.305)	1.168 (1.693)	-3.331 (16.706)	-2.000 (16.843)	1.334 (1.361)
LoP	30.064 (56.991)	52.296 (55.470)	5.001 (4.573)	2.574 (45.535)	10.892 (46.013)	1.657 (3.676)
NoL	191.963 (213.357)	139.016 (208.887)	-11.035 (17.243)	297.551 (170.469) (*)	259.525 (173.273)	-6.465 (13.860)
Age	-55.487 (12.951) ***	-46.439 (12.507) ***	2.073 (1.032) *	-41.815 (10.348) ***	-35.444 (10.375) ***	1.300 (0.830)
Sex (males = 0, females = 1)	-643.802 (368.078)	-477.457 (361.293)	30.559 (30.231)	-504.790 (294.089)	-253.690 (299.695)	67.408 (24.300) **
Education	-51.761 (93.146)	-37.032 (90.690)	0.740 (7.417)	-52.370 (74.422)	-29.150 (75.228)	2.976 (5.962)
ICV	8.144 (1.333) ***	8.151 (1.319) ***	-0.045 (0.107)	6.291 (1.065) ***	6.674 (1.094) ***	0.127 (0.086)
Time interval	-	-	-16.188 (15.432)	-	-	-13.220 (12.404)
Constant	5756.813 (2461.246) *	4769.975 (2482.168)	-150.413 (209.681)	6089.380 (1966.498) **	4741.196 (2058.978) *	-354.015 (168.539) *

(*)$p = 0.084$; *$p < 0.05$; **$p < 0.01$; ***$p < 0.001$.
Key: GMV, gray matter volume; IPL, inferior parietal lobule; AoA, age of acquisition; LoP, level of proficiency; NoL, number of actively spoken languages; ICV, intracranial volume; B, unstandardized coefficient B; SE, standard error.

Supplementary Table 12. Results for regression analyses for CT in the IFG as dependent variable for bilinguals of the total sample (n = 113)

	Left IFG			Right IFG		
	CT at t1	CT at t2	CT difference	CT at t1	CT at t2	CT difference
General model statistics	$R^2 = 0.227$<br>F[7, 105] = 4.410<br>p < 0.001***	$R^2 = 0.160$<br>F[7, 105] = 2.849<br>p = 0.009 **	$R^2 = 0.124$<br>F[8, 104] = 1.834<br>p = 0.079	$R^2 = 0.232$<br>F[7, 105] = 4.521<br>p < 0.001***	$R^2 = 0.216$<br>F[7, 105] = 4.143<br>p < 0.001***	$R^2 = 0.083$<br>F[8, 104] = 1.183<br>p = 0.316
**Predictors**	**B (SE)**	**B (SE)**	**B (SE)**	**B (SE)**	**B (SE)**	**B (SE)**
AoA	-0.003<br>(0.002) (*[1])	-0.003<br>(0.002)	< 0.001<br>(< 0.001)	0.001<br>(0.002)	0.001<br>(0.002)	< 0.001<br>(< 0.001)
LoP	0.001 (0.005)	0.005 (0.005)	0.002 (0.001) *	-0.001 (0.004)	0.002 (0.005)	0.001 (0.001) (*[2])
NoL	0.005 (0.018)	-0.004 (0.019)	-0.004 (0.003)	0.006 (0.016)	0.013 (0.017)	0.001 (0.002)
Age	-0.004<br>(0.001) ***	-0.003<br>(0.001) *	0.001<br>(< 0.001) **	-0.005<br>(0.001) ***	- 0.004<br>(0.001) ***	< 0.001<br>(< 0.001)
Sex (males = 0, females = 1)	0.020 (0.031)	0.033 (0.032)	0.007 (0.005)	0.013 (0.028)	0.034 (0.030)	0.009 (0.004) *
Education	0.004 (0.008)	0.004 (0.008)	< 0.001 (0.001)	0.015 (0.007) *	0.010 (0.008)	-0.001 (0.001)
ICV	-1.813×10^{-5}<br>(< 0.001)	-6.866×10^{-6}<br>(< 0.001)	< 0.001<br>(< 0.001)	-9.323×10^{-5}<br>(< 0.001)	-7.067×10^{-5}<br>(< 0.001)	< 0.001<br>(< 0.001)
Time interval	-	-	0.002 (0.002)	-	-	0.001 (0.002)
Constant	2.967 (0.208) ***	2.814 (0.221) ***	-0.075 (0.033) *	2.898 (0.188) ***	2.848 (0.207) ***	-0.039 (0.029)

(*[1])*p* = 0.054; (*[2])*p* = 0.091; *p < 0.05; **p < 0.01; ***p < 0.001.
Key: CT, cortical thickness; IFG, inferior frontal gyrus; AoA, age of acquisition; LoP, level of proficiency; NoL, number of actively spoken languages; ICV, intracranial volume; B, unstandardized coefficient B; SE, standard error.

Supplementary Table 13. Results for regression analyses for CT in the IPL as dependent variable for bilinguals of the total sample (n = 113)

	Left IPL			Right IPL		
	CT at t1	CT at t2	CT difference	CT at t1	CT at t2	CT difference
General model statistics	$R^2 = 0.395$<br>F[7, 105] = 9.813<br>p < 0.001***	$R^2 = 0.365$<br>F[7, 105] = 8.621<br>p < 0.001 ***	$R^2 = 0.080$<br>F[8, 104] = 1.137<br>p = 0.345	$R^2 = 0.346$<br>F[7, 105] = 7.939<br>p < 0.001***	$R^2 = 0.304$<br>F[7, 105] = 6.567<br>p < 0.001***	$R^2 = 0.074$<br>F[8, 104] = 1.038<br>p = 0.413
**Predictors**	**B (SE)**	**B (SE)**	**B (SE)**	**B (SE)**	**B (SE)**	**B (SE)**
AoA	-0.001 (0.001)	-0.001 (0.001)	< 0.001 (< 0.001)	-0.001 (0.002)	< 0.001 (0.002)	< 0.001 (< 0.001)
LoP	-0.002 (0.004)	0.002 (0.004)	0.001 (0.001) (*)	-0.004 (0.004)	-0.001 (0.004)	0.001 (0.001)
NoL	0.022 (0.014)	0.014 (0.015)	-0.003 (0.003)	0.021 (0.016)	0.015 (0.016)	-0.002 (0.003)
Age	-0.006<br>(0.001) ***	-0.005<br>(0.001) ***	< 0.001<br>(< 0.001) *	-0.006<br>(0.001) ***	-0.006<br>(0.001) ***	< 0.001<br>(< 0.001)
Sex (males = 0, females = 1)	0.008 (0.025)	0.028 (0.026)	0.007 (0.005)	-0.040 (0.027)	-0.005 (0.027)	0.012 (0.005) *
Education	-0.001 (0.006)	0.001 (0.006)	0.001 (0.001)	0.009 (0.007)	0.007 (0.007)	< 0.001 (0.001)
ICV	< 0.001<br>(< 0.001)	< 0.001<br>(< 0.001)	-1.262×10^{-7}<br>(< 0.001)	< 0.001<br>(< 0.001)	-6.980×10^{-5}<br>(< 0.001)	< 0.001<br>(< 0.001)
Time interval	-	-	< 0.001 (0.002)	-	-	< 0.001 (0.002)
Constant	3.193 (0.167) ***	3.090 (0.176) ***	-0.044 (0.032)	3.116 (0.182) ***	2.960 (0.189) ***	-0.064 (0.034)

(*)*p* = 0.055; **p* < 0.05; ***p* < 0.01; ****p* < 0.001.
Key: CT, cortical thickness; IPL, inferior parietal lobule; AoA, age of acquisition; LoP, level of proficiency; NoL, number of actively spoken languages; ICV, intracranial volume; B, unstandardized coefficient B; SE, standard error.

Supplementary Table 14. Results for regression analyses for SA in the IFG as dependent variable for bilinguals of the total sample (n = 113)

	Left IFG			Right IFG		
	SA at t1	SA at t2	SA difference	SA at t1	SA at t2	SA difference
General model statistics	R^2 = 0.344<br>F[7, 105] = 7.880<br>p < 0.001***	R^2 = 0.350<br>F[7, 105] = 8.084<br>p < 0.001***	R^2 = 0.163<br>F[8, 104] = 2.535<br>p = 0.015 *	R^2 = 0.427<br>F[7, 105] = 11.170<br>p < 0.001***	R^2 = 0.420<br>F[7, 105] = 10.850<br>p < 0.001***	R^2 = 0.031<br>F[8, 104] = 0.419<br>p = 0.907
**Predictors**	**B (SE)**	**B (SE)**	**B (SE)**	**B (SE)**	**B (SE)**	**B (SE)**
AoA	3.118 (1.597) (*)	3.654 (1.607) *	0.221 (0.105) *	0.786 (2.064)	0.263 (2.069)	-0.050 (0.140)
LoP	-2.783 (4.352)	-3.241 (4.391)	-0.300 (0.282)	1.604 (5.625)	1.637 (5.653)	-0.297 (0.377)
NoL	-4.472 (16.293)	-6.484 (16.534)	0.923 (1.065)	-30.627 (21.058)	-32.190 (21.288)	0.558 (1.421)
Age	-2.596 (0.989) **	-2.809 (0.990) **	-0.084 (0.064)	-3.750 (1.278) **	-3.496 (1.275) **	-0.002 (0.085)
Sex(males = 0, females = 1)	-43.348 (28.109)	-29.761 (28.598)	0.210 (1.867)	-28.838 (36.328)	-27.616 (36.820)	-1.420 (2.492)
Education	3.183 (7.113)	5.151 (7.179)	-0.129 (0.458)	-3.409 (9.193)	-0.392 (9.243)	0.397 (0.611)
ICV	0.470 (0.102) ***	0.500 (0.104) ***	-0.002 (0.007)	0.798 (0.132) ***	0.784 (0.134) ***	-0.007 (0.009)
Time interval	-	-	-3.507 (0.953) ***	-	-	-1.722 (1.272)
Constant	706.843 (187.957) ***	650.561 (196.476) **	15.246 (12.951)	538.134 (242.917) *	525.697 (252.965) *	12.560 (17.283)

(*)p = 0.054; *p < 0.05; **p < 0.01; ***p < 0.001.
Key: SA, surface area; IFG, inferior frontal gyrus; AoA, age of acquisition; LoP, level of proficiency; NoL, number of actively spoken languages; ICV, intracranial volume; B, unstandardized coefficient B; SE, standard error.

Supplementary Table 15. Results for regression analyses for SA in the IPL as dependent variable for bilinguals of the total sample (n = 113)

	Left IPL			Right IPL		
	SA at t1	SA at t2	SA difference	SA at t1	SA at t2	SA difference
General model statistics	$R^2 = 0.514$ $F[7, 105] = 15.852$ $p < 0.001$***	$R^2 = 0.511$ $F[7, 105] = 15.656$ $p < 0.001$***	$R^2 = 0.019$ $F[8, 104] = 0.252$ $p = 0.979$	$R^2 = 0.461$ $F[7, 105] = 12.820$ $p < 0.001$***	$R^2 = 0.458$ $F[7, 105] = 12.694$ $p < 0.001$***	$R^2 = 0.035$ $F[8, 104] = 0.466$ $p = 0.877$
**Predictors**	**B (SE)**	**B (SE)**	**B (SE)**	**B (SE)**	**B (SE)**	**B (SE)**
AoA	-1.845 (6.799)	-2.611 (6.820)	0.071 (0.280)	-1.485 (5.628)	-1.970 (5.592)	0.057 (0.389)
LoP	9.627 (18.532)	11.083 (18.632)	-0.318 (0.756)	4.492 (15.340)	2.902 (15.276)	-0.977 (1.051)
NoL	24.384 (69.379)	20.924 (70.163)	1.802 (2.852)	63.366 (57.428)	57.148 (57.525)	0.672 (3.963)
Age	-4.982 (4.211)	-3.921 (4.201)	-0.043 (0.171)	-0.444 (3.486)	0.799 (3.444)	0.125 (0.237)
Sex (males = 0, females = 1)	-215.593 (119.691)	-223.347 (121.355)	-5.871 (5.000)	-89.434 (99.073)	-82.701 (99.496)	-0.964 (6.948)
Education	1.834 (30.289)	4.177 (30.462)	-0.911 (1.227)	-38.310 (25.071)	-23.547 (24.975)	2.367 (1.705)
ICV	3.096 (0.434) ***	3.076 (0.443) ***	-0.009 (0.018)	2.570 (0.359) ***	2.561 (0.363) ***	-0.001 (0.025)
Time interval	-	-	-1.812 (2.552)	-	-	-0.767 (3.546)
Constant	584.338 (800.341)	519.569 (833.740)	19.902 (34.682)	860.862 (662.475)	697.008 (683.558)	-19.320 (48.188)

*$p < 0.05$; **$p < 0.01$; ***$p < 0.001$.
Key: SA, surface area; IPL, inferior parietal lobule; AoA, age of acquisition; LoP, level of proficiency; NoL, number of actively spoken languages; ICV, intracranial volume; B, unstandardized coefficient B; SE, standard error.

Supplementary Table 16. Results for regression analyses for GMV in the IFG as dependent variable for bilinguals of the older subsample (n = 71)

	Left IFG			Right IFG		
	GMV at t1	GMV at t2	GMV difference	GMV at t1	GMV at t2	GMV difference
General model statistics	$R^2 = 0.232$<br>F[7, 63] = 2.724<br>p = 0.016 *	$R^2 = 0.277$<br>F[7, 63] = 3.447<br>p = 0.003 **	$R^2 = 0.071$<br>F[8, 62] = 0.590<br>p = 0.782	$R^2 = 0.298$<br>F[7, 63] = 3.822<br>p = 0.002 **	$R^2 = 0.314$<br>F[7, 63] = 4.119<br>p < 0.001 ***	$R^2 = 0.138$<br>F[8, 62] = 1.237<br>p = 0.293
**Predictors**	**B (SE)**	**B (SE)**	**B (SE)**	**B (SE)**	**B (SE)**	**B (SE)**
AoA	7.461 (6.416)	10.760 (5.956) (*1)	0.897 (0.530) (*2)	5.580 (7.392)	5.272 (7.082)	0.316 (0.400)
LoP	-2.264 (19.502)	-0.737 (18.085)	0.410 (1.631)	14.093 (22.469)	17.885 (21.505)	0.751 (1.230)
NoL	-5.965 (73.334)	-31.362 (67.851)	-4.939 (6.143)	-75.164 (84.488)	-58.606 (80.680)	6.302 (4.632)
Age	-8.201 (8.630)	-7.834 (8.028)	-0.274 (0.706)	-14.087 (9.942)	-10.772 (9.546)	-0.116 (0.532)
Sex (males = 0, females = 1)	-0.092 (129.456)	81.363 (122.327)	8.492 (11.279)	72.193 (149.147)	138.278 (145.454)	9.211 (8.506)
Education	7.430 (31.429)	23.017 (28.990)	2.937 (2.574)	20.882 (36.210)	7.013 (34.471)	-2.606 (1.941)
ICV	1.643 (0.467) ***	1.809 (0.442) ***	0.005 (0.038)	2.261 (0.538) ***	2.422 (0.525) ***	0.011 (0.029)
Time interval	-	-	-1.860 (5.587)	-	-	-4.737 (4.214)
Constant	1972.983 (985.861) *	1497.861 (958.748)	-35.096 (85.394)	1610.394 (1135.822)	1151.965 (1140.011)	-15.162 (64.401)

(*1)$p = 0.076$; (*2)$p = 0.096$; *$p < 0.05$; **$p < 0.01$; ***$p < 0.001$.
Key: GMV, gray matter volume; IFG, inferior frontal gyrus; AoA, age of acquisition; LoP, level of proficiency; NoL, number of actively spoken languages; ICV, intracranial volume; B, unstandardized coefficient B; SE, standard error.

Supplementary Table 17. Results for regression analyses for GMV in the IPL as dependent variable for bilinguals of the older subsample (n = 71)

	Left IPL			Right IPL		
	GMV at t1	GMV at t2	GMV difference	GMV at t1	GMV at t2	GMV difference
General model statistics	$R^2 = 0.437$ F[7, 63] = 6.974 p < 0.001 ***	$R^2 = 0.448$ F[7, 63] = 7.316 p < 0.001 ***	$R^2 = 0.054$ F[8, 62] = 0.440 p = 0.893	$R^2 = 0.507$ F[7, 63] = 9.270 p < 0.001 ***	$R^2 = 0.512$ F[7, 63] = 9.443 p < 0.001 ***	$R^2 = 0.135$ F[8, 62] = 1.208 p = 0.309
**Predictors**	**B (SE)**	**B (SE)**	**B (SE)**	**B (SE)**	**B (SE)**	**B (SE)**
AoA	-29.135 (22.719)	-27.188 (21.365)	1.514 (1.765)	-4.014 (17.721)	0.603 (17.222)	2.045 (1.320)
LoP	2.452 (69.059)	18.566 (64.873)	4.606 (5.427)	-10.174 (53.867)	0.063 (52.294)	3.046 (4.058)
NoL	147.013 (259.677)	106.755 (243.385)	-5.775 (20.441)	245.184 (202.552)	244.453 (196.195)	5.643 (15.284)
Age	-62.352 (30.558) *	-62.115 (28.797) *	-1.641 (2.348)	-53.863 (23.836) *	-53.719 (23.214) *	-1.650 (1.756)
Sex (males = 0, females = 1)	-314.368 (458.408)	-145.811 (438.790)	2.845 (37.532)	-297.361 (357.564)	-16.743 (353.713)	53.436 (28.064)
Education	-80.156 (111.291)	-50.479 (103.989)	4.029 (8.565)	-93.585 (86.809)	-64.142 (83.826)	6.470 (6.405)
ICV	7.687 (1.654) ***	7.687 (1.584) ***	-0.133 (0.127)	7.276 (1.290) ***	7.680 (1.277) ***	0.051 (0.095)
Time interval	-	-	-7.753 (18.593)	-	-	-5.106 (13.902)
Constant	7687.090 (3490.979) *	7208.087 (3439.054) *	166.286 (284.168)	5913.669 (2723.009) *	4794.172 (2772.253)	-145.409 (212.479)

*$p < 0.05$; **$p < 0.01$; ***$p < 0.001$.

Key: GMV, gray matter volume; IPL, inferior parietal lobule; AoA, age of acquisition; LoP, level of proficiency; NoL, number of actively spoken languages; ICV, intracranial volume; B, unstandardized coefficient B; SE, standard error.

Supplementary Table 18. Results for regression analyses for CT in the IFG as dependent variable for bilinguals of the older subsample (n = 71)

	Left IFG			Right IFG		
	CT at t1	CT at t2	CT difference	CT at t1	CT at t2	CT difference
General model statistics	$R^2 = 0.059$<br>F[7, 63] = 0.569<br>p = 0.778	$R^2 = 0.061$<br>F[7, 63] = 0.584<br>p = 0.766	$R^2 = 0.045$<br>F[8, 62] = 0.362<br>p = 0.937	$R^2 = 0.144$<br>F[7, 63] = 1.515<br>p = 0.179	$R^2 = 0.150$<br>F[7, 63] = 1.588<br>p = 0.156	$R^2 = 0.104$<br>F[8, 62] = 0.900<br>p = 0.522
**Predictors**	**B (SE)**	**B (SE)**	**B (SE)**	**B (SE)**	**B (SE)**	**B (SE)**
AoA	-0.003 (0.002)	-0.002 (0.002)	< 0.001 (< 0.001)	0.002 (0.002)	0.003 (0.002)	< 0.001 (< 0.001)
LoP	0.001 (0.006)	0.004 (0.006)	0.001 (0.001)	0.005 (0.005)	0.008 (0.006)	0.001 (0.001)
NoL	0.016 (0.023)	0.005 (0.023)	-0.004 (0.003)	0.003 (0.019)	0.013 (0.021)	0.002 (0.003)
Age	0.001 (0.003)	0.001 (0.003)	< 0.001 (< 0.001)	-0.003 (0.002)	-0.003 (0.003)	-6.191×10^{-5} (< 0.001)
Sex (males = 0, females = 1)	0.039 (0.041)	0.050 (0.042)	0.005 (0.006)	0.043 (0.033)	0.066 (0.039)	0.010 (0.006)
Education	0.003 (0.010)	0.006 (0.010)	0.001 (0.001)	0.019 (0.008) *	0.013 (0.009)	-0.001 (0.001)
ICV	< 0.001 (< 0.001)	< 0.001 (< 0.001)	< 0.001 (< 0.001)	< 0.001 (< 0.001)	< 0.001 (< 0.001)	< 0.001 (< 0.001)
Time interval	-	-	0.003 (0.003)	-	-	0.002 (0.003)
Constant	2.483 (0.315) ***	2.455 (0.327) ***	-0.035 (0.046)	2.504 (0.255) ***	2.455 (0.304) ***	-0.043 (0.047)

*$p < 0.05$; **$p < 0.01$; ***$p < 0.001$.
Key: CT, cortical thickness; IFG, inferior frontal gyrus; AoA, age of acquisition; LoP, level of proficiency; NoL, number of actively spoken languages; ICV, intracranial volume; B, unstandardized coefficient B; SE, standard error.

Supplementary Table 19. Results for regression analyses for CT in the IPL as dependent variable for bilinguals of the older subsample (n = 71)

	Left IPL			Right IPL		
	CT at t1	CT at t2	CT difference	CT at t1	CT at t2	CT difference
General model statistics	R^2 = 0.206<br>F[7, 63] = 2.331<br>p = 0.035 *	R^2 = 0.238<br>F[7, 63] = 2.816<br>p = 0.013 *	R^2 = 0.060<br>F[8, 62] = 0.496<br>p = 0.855	R^2 = 0.184<br>F[7, 63] = 2.032<br>p = 0.065	R^2 = 0.192<br>F[7, 63] = 2.140<br>p = 0.052	R^2 = 0.058<br>F[8, 62] = 0.476<br>p = 0.868
**Predictors**	**B (SE)**	**B (SE)**	**B (SE)**	**B (SE)**	**B (SE)**	**B (SE)**
AoA	-0.001 (0.002)	-0.001 (0.001)	< 0.001 (< 0.001)	-0.001 (0.002)	< 0.001 (0.002)	< 0.001 (< 0.001)
LoP	-0.001 (0.005)	0.001 (0.005)	0.001 (0.001)	-0.003 (0.005)	< 0.001 (0.005)	0.001 (0.001)
NoL	0.031 (0.017) (*[1])	0.023 (0.017)	-0.003 (0.003)	0.035 (0.019) (*[2])	0.035 (0.018) (*[3])	-0.001 (0.004)
Age	-0.005 (0.002) *	-0.006 (0.002) **	< 0.001 (< 0.001)	-0.006 (0.002) **	-0.007 (0.002) **	< 0.001 (< 0.001)
Sex (males = 0, females = 1)	0.030 (0.031)	0.037 (0.031)	0.003 (0.006)	-0.045 (0.034)	-0.015 (0.033)	0.010 (0.007)
Education	< 0.001 (0.007)	0.003 (0.007)	0.001 (0.001)	0.005 (0.008)	0.004 (0.008)	< 0.001 (0.002)
ICV	< 0.001 (< 0.001)	< 0.001 (< 0.001)	-1.310×10^{-5} (< 0.001)	< 0.001 (< 0.001)	-8.825×10^{-5} (< 0.001)	< 0.001 (< 0.001)
Time interval	-	-	0.002 (0.003)	-	-	0.001 (0.003)
Constant	3.102 (0.233) ***	3.160 (0.239) ***	0.010 (0.043)	3.110 (0.260) ***	3.017 (0.256) ***	-0.034 (0.051)

(*[1])*p* = 0.083; (*[2])*p* = 0.076; (*[3])*p* = 0.059; **p* < 0.05; ***p* < 0.01; ****p* < 0.001.
Key: CT, cortical thickness; IPL, inferior parietal lobule; AoA, age of acquisition; LoP, level of proficiency; NoL, number of actively spoken languages; ICV, intracranial volume; B, unstandardized coefficient B; SE, standard error.

Supplementary Table 20. Results for regression analyses for SA in the IFG as dependent variable for bilinguals of the older subsample (n = 71)

	Left IFG			Right IFG		
	SA at t1	SA at t2	SA difference	SA at t1	SA at t2	SA difference
General model statistics	$R^2 = 0.323$<br>F[7, 63] = 4.293<br>p < 0.001 ***	$R^2 = 0.345$<br>F[7, 63] = 4.736<br>p < 0.001 ***	$R^2 = 0.219$<br>F[8, 62] = 2.175<br>p = 0.042 *	$R^2 = 0.324$<br>F[7, 63] = 4.311<br>p < 0.001 ***	$R^2 = 0.332$<br>F[7, 63] = 4.475<br>p < 0.001 ***	$R^2 = 0.061$<br>F[8, 62] = 0.504<br>p = 0.849
**Predictors**	**B (SE)**	**B (SE)**	**B (SE)**	**B (SE)**	**B (SE)**	**B (SE)**
AoA	4.152 (1.897) *	4.905 (1.861) *	0.277 (0.118) *	0.433 (2.272)	< 0.001 (2.227)	-0.003 (0.181)
LoP	-3.137 (5.765)	-3.491 (5.651)	-0.426 (0.363)	1.054 (6.907)	0.838 (6.763)	-0.502 (0.556)
NoL	-15.566 (21.679)	-17.527 (21.200)	1.186 (1.368)	-31.534 (25.973)	-34.116 (25.374)	0.031 (2.093)
Age	-3.162 (2.551)	-3.209 (2.508)	-0.134 (0.157)	-2.054 (3.056)	-1.071 (3.002)	-0.074 (0.240)
Sex (males = 0, females = 1)	-22.586 (38.270)	-3.216 (38.221)	-1.487 (2.513)	2.664 (45.850)	6.273 (45.745)	-4.256 (3.842)
Education	5.814 (9.291)	7.515 (9.058)	-0.094 (0.573)	-3.953 (11.131)	-2.478 (10.841)	0.565 (0.877)
ICV	0.538 (0.138) ***	0.577 (0.138) ***	-0.008 (0.008)	0.742 (0.165) ***	0.756 (0.165) ***	-0.010 (0.013)
Time interval	-	-	-3.844 (1.245) **	-	-	-2.761 (1.903)
Constant	617.856 (291.444) *	532.759 (299.563)	29.557 (19.024)	516.845 (349.165)	419.432 (358.532)	26.838 (29.090)

*$p < 0.05$; **$p < 0.01$; ***$p < 0.001$.

Key: SA, surface area; IFG, inferior frontal gyrus; AoA, age of acquisition; LoP, level of proficiency; NoL, number of actively spoken languages; ICV, intracranial volume; B, unstandardized coefficient B; SE, standard error.

Supplementary Table 21. Results for regression analyses for SA in the IPL as dependent variable for bilinguals of the older subsample (n = 71)

	Left IPL			Right IPL		
	SA at t1	SA at t2	SA difference	SA at t1	SA at t2	SA difference
General model statistics	$R^2 = 0.498$<br>F[7, 63] = 8.932<br>p < 0.001 ***	$R^2 = 0.492$<br>F[7, 63] = 8.731<br>p < 0.001 ***	$R^2 = 0.047$<br>F[8, 62] = 0.381<br>p = 0.927	$R^2 = 0.475$<br>F[7, 63] = 8.149<br>p < 0.001 ***	$R^2 = 0.473$<br>F[7, 63] = 8.088<br>p < 0.001 ***	$R^2 = 0.043$<br>F[8, 62] = 0.346<br>p = 0.944
**Predictors**	**B (SE)**	**B (SE)**	**B (SE)**	**B (SE)**	**B (SE)**	**B (SE)**
AoA	-8.219 (7.554)	-8.884 (7.498)	0.082 (0.313)	-2.322 (6.441)	-2.631 (6.347)	0.144 (0.514)
LoP	-4.610 (22.961)	-3.148 (22.766)	-0.048 (0.963)	-1.704 (19.578)	-3.908 (19.271)	-1.265 (1.579)
NoL	14.257 (86.338)	16.599 (85.413)	2.769 (3.626)	27.098 (73.617)	20.076 (72.300)	0.573 (5.948)
Age	-10.184 (10.160)	-9.773 (10.106)	-0.379 (0.417)	-4.453 (8.663)	-3.780 (8.554)	-0.153 (0.683)
Sex (males = 0, females = 1)	-214.813 (152.412)	-194.440 (153.988)	-4.830 (6.657)	-13.474 (129.956)	5.867 (130.347)	-4.728 (10.921)
Education	-7.969 (37.002)	-2.260 (36.494)	-0.459 (1.519)	-46.980 (31.550)	-31.466 (30.891)	3.310 (2.492)
ICV	2.997 (0.550) ***	2.951 (0.556) ***	-0.020 (0.022)	2.915 (0.469) ***	2.923 (0.471) ***	-0.008 (0.037)
Time interval	-	-	-3.862 (3.298)	-	-	-1.983 (5.410)
Constant	1419.837 (1160.686)	1400.963 (1206.894)	57.973 (50.404)	794.031 (989.668)	646.805 (1021.606)	7.902 (82.689)

*$p < 0.05$; **$p < 0.01$; ***$p < 0.001$.

Key: SA, surface area; IPL, inferior parietal lobule; AoA, age of acquisition; LoP, level of proficiency; NoL, number of actively spoken languages; ICV, intracranial volume; B, unstandardized coefficient B; SE, standard error.

Supplementary Table 22. Results for regression analyses for GMV in the IFG as dependent variable for bilinguals of the total sample (n = 113) excluding ICV as predictor

	Left IFG			Right IFG		
	GMV at t1	GMV at t2	GMV difference	GMV at t1	GMV at t2	GMV difference
General model statistics	$R^2 = 0.269$ $F[6, 106] = 6.487$ $p < 0.001$ ***	$R^2 = 0.234$ $F[6, 106] = 5.403$ $p < 0.001$ ***	$R^2 = 0.120$ $F[7, 105] = 2.037$ $p = 0.057$	$R^2 = 0.328$ $F[6, 106] = 8.638$ $p < 0.001$ ***	$R^2 = 0.316$ $F[6, 106] = 8.161$ $p < 0.001$ ***	$R^2 = 0.134$ $F[7, 105] = 2.328$ $p = 0.030$ *
**Predictors**	**B (SE)**	**B (SE)**	**B (SE)**	**B (SE)**	**B (SE)**	**B (SE)**
AoA	1.442 (5.848)	4.478 (5.782)	0.747 (0.481)	0.132 (7.276)	-0.674 (7.208)	-0.015 (0.375)
LoP	-9.657 (15.897)	-6.784 (15.696)	1.495 (1.289)	-5.398 (19.778)	-2.868 (19.565)	1.161 (1.006)
NoL	33.448 (59.343)	18.429 (58.943)	-5.367 (4.841)	-1.315 (73.832)	9.969 (73.472)	2.299 (3.779)
Age	-18.381 (3.609) ***	-16.530 (3.523) ***	0.790 (0.292) **	-24.635 (4.491) ***	-24.016 (4.391) ***	0.294 (0.228)
Sex (males = 0, females = 1)	-294.898 (86.005) ***	-253.010 (84.893) **	12.010 (7.073)	-437.434 (107.002) ***	-393.123 (105.818) ***	15.012 (5.521) **
Education	11.476 (26.126)	19.144 (25.879)	1.633 (2.108)	14.722 (32.504)	6.018 (32.258)	-1.732 (1.645)
Time interval	-	-	-3.371 (4.336)	-	-	-5.337 (3.384)
Constant	5269.430 (322.757) ***	5026.058 (326.844) ***	-87.740 (29.362) **	6032.183 (401.557) ***	6031.939 (407.406) ***	-23.348 (22.918)

**p* < 0.05; ***p* < 0.01; ****p* < 0.001.
Key: GMV, gray matter volume; IFG, inferior frontal gyrus; AoA, age of acquisition; LoP, level of proficiency; NoL, number of actively spoken languages; ICV, intracranial volume; B, unstandardized coefficient B; SE, standard error.

Supplementary Table 23. Results for regression analyses for GMV in the IPL as dependent variable for bilinguals of the total sample (n = 113) excluding ICV as predictor

	Left IPL			Right IPL		
	GMV at t1	GMV at t2	GMV difference	GMV at t1	GMV at t2	GMV difference
General model statistics	$R^2 = 0.333$ F[6, 106] = 8.816 p < 0.001 ***	$R^2 = 0.316$ F[6, 106] = 8.149 p < 0.001 ***	$R^2 = 0.073$ F[7, 105] = 1.178 p = 0.321	$R^2 = 0.310$ F[6, 106] = 7.932 p < 0.001 ***	$R^2 = 0.264$ F[6, 106] = 6.335 p < 0.001 ***	$R^2 = 0.093$ F[7, 105] = 1.544 p = 0.160
**Predictors**	**B (SE)**	**B (SE)**	**B (SE)**	**B (SE)**	**B (SE)**	**B (SE)**
AoA	-21.527 (24.148)	-19.830 (23.551)	1.203 (1.685)	-11.300 (19.128)	-8.713 (19.469)	1.233 (1.367)
LoP	-8.007 (65.638)	7.652 (63.927)	5.243 (4.518)	-26.836 (51.994)	-25.664 (52.847)	0.967 (3.667)
NoL	364.770 (245.025)	332.145 (240.069)	-12.153 (16.965)	431.047 (194.092) *	417.662 (198.457) *	-3.281 (13.767)
Age	-64.721 (14.903) ***	-58.909 (14.348) ***	2.118 (1.023) *	-48.948 (11.805) ***	-45.655 (11.861) ***	1.171 (0.830)
Sex (males = 0, females = 1)	-1888.806 (355.108) ***	-1745.003 (345.762) ***	37.705 (24.785)	-1466.574 (281.292) ***	-1291.577 (285.830) ***	47.056 (20.112) *
Education	-33.846 (107.871)	-28.902 (105.403)	0.666 (7.386)	-38.531 (85.448)	-22.493 (87.134)	3.188 (5.993)
Time interval	-	-	-15.214 (15.193)	-	-	-15.994 (12.329)
Constant	19047.093 (1332.643) ***	18382.950 (1331.200) ***	-226.360 (102.891) *	16356.318 (1055.628) ***	15887.724 (1100.461) ***	-137.705 (83.493)

**p* < 0.05; ***p* < 0.01; ****p* < 0.001.
Key: GMV, gray matter volume; IPL, inferior parietal lobule; AoA, age of acquisition; LoP, level of proficiency; NoL, number of actively spoken languages; ICV, intracranial volume; B, unstandardized coefficient B; SE, standard error.

Supplementary Table 24. Results for regression analyses for CT in the IFG as dependent variable for bilinguals of the total sample (n = 113) excluding ICV as predictor

	Left IFG			Right IFG		
	CT at t1	CT at t2	CT difference	CT at t1	CT at t2	CT difference
General model statistics	$R^2 = 0.227$<br>F[6, 106] = 5.189<br>p < 0.001 ***	$R^2 = 0.160$<br>F[6, 106] = 3.355<br>p = 0.005 **	$R^2 = 0.119$<br>F[7, 105] = 2.034<br>p = 0.058	$R^2 = 0.225$<br>F[6, 106] = 5.143<br>p < 0.001 ***	$R^2 = 0.213$<br>F[6, 106] = 4.791<br>p < 0.001 ***	$R^2 = 0.079$<br>F[7, 105] = 1.283<br>p = 0.266
**Predictors**	**B (SE)**	**B (SE)**	**B (SE)**	**B (SE)**	**B (SE)**	**B (SE)**
AoA	-0.003 (0.002) (*)	-0.003 (0.002)	< 0.001 (< 0.001)	0.001 (0.002)	0.001 (0.002)	< 0.001 (< 0.001)
LoP	0.001 (0.005)	0.005 (0.005)	0.001 (0.001) *	< 0.001 (0.004)	0.002 (0.005)	0.001 (0.001)
NoL	0.005 (0.018)	-0.004 (0.018)	-0.004 (0.003)	0.004 (0.016)	0.011 (0.017)	0.002 (0.002)
Age	-0.004<br>(0.001) ***	-0.003<br>(0.001) *	0.001<br>(< 0.001) **	-0.005<br>(0.001) ***	-0.004<br>(0.001) ***	< 0.001<br>(< 0.001)
Sex (males = 0, females = 1)	0.023 (0.026)	0.034 (0.026)	0.005 (0.004)	0.027 (0.023)	0.045 (0.025)	0.007 (0.003) *
Education	0.004 (0.008)	0.004 (0.008)	< 0.001 (0.001)	0.015 (0.007) *	0.010 (0.008)	-0.001 (0.001)
Time interval	-	-	0.002 (0.002)	-	-	0.001 (0.002)
Constant	2.938<br>(0.097) ***	2.803<br>(0.101) ***	-0.055<br>(0.016) ***	2.746<br>(0.088) ***	2.730<br>(0.095) ***	-0.021<br>(0.014)

(*)*p* = 0.054; **p* < 0.05; ***p* < 0.01; ****p* < 0.001.
Key: CT, cortical thickness; IFG, inferior frontal gyrus; AoA, age of acquisition; LoP, level of proficiency; NoL, number of actively spoken languages; ICV, intracranial volume; B, unstandardized coefficient B; SE, standard error.

Supplementary Table 25. Results for regression analyses for CT in the IPL as dependent variable for bilinguals of the total sample (n = 113) excluding ICV as predictor

	Left IPL			Right IPL		
	CT at t1	CT at t2	CT difference	CT at t1	CT at t2	CT difference
General model statistics	$R^2 = 0.379$<br>F[6, 106] = 10.794<br>p < 0.001 ***	$R^2 = 0.348$<br>F[6, 106] = 9.435<br>p < 0.001 ***	$R^2 = 0.080$<br>F[7, 105] = 1.312<br>p = 0.252	$R^2 = 0.335$<br>F[6, 106] = 8.916<br>p < 0.001 ***	$R^2 = 0.301$<br>F[6, 106] = 7.618<br>p < 0.001 ***	$R^2 = 0.056$<br>F[7, 105] = 0.897<br>p = 0.512
**Predictors**	**B (SE)**	**B (SE)**	**B (SE)**	**B (SE)**	**B (SE)**	**B (SE)**
AoA	-0.001 (0.001)	-0.001 (0.001)	< 0.001 (< 0.001)	-0.001 (0.002)	< 0.001 (0.002)	< 0.001 (< 0.001)
LoP	-0.001 (0.004)	0.003 (0.004)	0.001 (0.001) (*)	-0.003 (0.004)	-0.001 (0.004)	0.001 (0.001)
NoL	0.019 (0.014)	0.011 (0.015)	-0.003 (0.003)	0.019 (0.016)	0.013 (0.016)	-0.002 (0.003)
Age	-0.006 (0.001) ***	-0.005 (0.001) ***	< 0.001 (< 0.001) *	-0.006 (0.001) ***	-0.006 (0.001) ***	< 0.001 (< 0.001)
Sex (males = 0, females = 1)	0.031 (0.021)	0.052 (0.021) *	0.007 (0.004)	-0.020 (0.023)	0.006 (0.023)	0.008 (0.004)
Education	-0.001 (0.006)	< 0.001 (0.006)	0.001 (0.001)	0.008 (0.007)	0.007 (0.007)	< 0.001 (0.001)
Time interval	-	-	< 0.001 (0.002)	-	-	-0.001 (0.002)
Constant	2.945 (0.079) ***	2.829 (0.082) ***	-0.044 (0.016) **	2.906 (0.085) ***	2.844 (0.087) ***	-0.023 (0.017)

(*)$p = 0.052$; *$p < 0.05$; **$p < 0.01$; ***$p < 0.001$.
Key: CT, cortical thickness; IPL, inferior parietal lobule; AoA, age of acquisition; LoP, level of proficiency; NoL, number of actively spoken languages; ICV, intracranial volume; B, unstandardized coefficient B; SE, standard error.

Supplementary Table 26. Results for regression analyses for SA in the IFG as dependent variable for bilinguals of the total sample (n = 113) excluding ICV as predictor

	Left IFG			Right IFG		
	SA at t1	SA at t2	SA difference	SA at t1	SA at t2	SA difference
General model statistics	$R^2 = 0.211$<br>F[6, 106] = 4.730<br>p < 0.001 ***	$R^2 = 0.208$<br>F[6, 106] = 4.646<br>p < 0.001 ***	$R^2 = 0.163$<br>F[7, 105] = 2.912<br>p = 0.008 **	$R^2 = 0.226$<br>F[6, 106] = 5.158<br>p < 0.001 ***	$R^2 = 0.231$<br>F[6, 106] = 5.319<br>p < 0.001 ***	$R^2 = 0.025$<br>F[7, 105] = 0.383<br>p = 0.911
**Predictors**	**B (SE)**	**B (SE)**	**B (SE)**	**B (SE)**	**B (SE)**	**B (SE)**
AoA	2.522 (1.738)	3.152 (1.762) (*)	0.222 (0.104) *	-0.226 (2.379)	-0.526 (2.365)	-0.044 (0.139)
LoP	-4.981 (4.723)	-5.979 (4.783)	-0.290 (0.279)	-2.128 (6.467)	-2.660 (6.420)	-0.257 (0.373)
NoL	5.508 (17.631)	5.361 (17.961)	0.878 (1.047)	-13.689 (24.140)	-13.602 (24.111)	0.375 (1.402)
Age	-3.130<br>(1.072) **	-3.574<br>(1.073) **	-0.082<br>(0.063)	-4.655<br>(1.468) **	-4.697<br>(1.441) **	0.006<br>(0.084)
Sex (males = 0, females = 1)	-115.250<br>(25.551) ***	-107.503<br>(25.868) ***	0.498<br>(1.530)	-150.869<br>(34.985) ***	-149.612<br>(34.726) ***	-0.247<br>(2.048)
Education	4.218 (7.762)	5.650 (7.886)	-0.132 (0.456)	-1.653 (10.627)	0.391 (10.586)	0.385 (0.610)
Time interval	-	-	-3.467<br>(0.938) ***	-	-	-1.562<br>(1.255)
Constant	1474.384<br>(95.889) ***	1485.479<br>(99.595) ***	12.192<br>(6.352)	1840.803<br>(131.291) ***	1835.889<br>(133.695) ***	0.099<br>(8.501)

(*)*p* = 0.077; **p* < 0.05; ***p* < 0.01; ****p* < 0.001.
Key: SA, surface area; IFG, inferior frontal gyrus; AoA, age of acquisition; LoP, level of proficiency; NoL, number of actively spoken languages; ICV, intracranial volume; B, unstandardized coefficient B; SE, standard error.

Supplementary Table 27. Results for regression analyses for SA in the IPL as dependent variable for bilinguals of the total sample (n = 113) excluding ICV as predictor

	Left IPL			Right IPL		
	SA at t1	SA at t2	SA difference	SA at t1	SA at t2	SA difference
General model statistics	$R^2 = 0.278$<br>F[6, 106] = 6.790<br>p < 0.001 ***	$R^2 = 0.286$<br>F[6, 106] = 7.075<br>p < 0.001 ***	$R^2 = 0.017$<br>F[7, 105] = 0.255<br>p = 0.970	$R^2 = 0.198$<br>F[6, 106] = 4.349<br>p < 0.001 ***	$R^2 = 0.202$<br>F[6, 106] = 4.466<br>p < 0.001 ***	$R^2 = 0.035$<br>F[7, 105] = 0.538<br>p = 0.804
**Predictors**	**B (SE)**	**B (SE)**	**B (SE)**	**B (SE)**	**B (SE)**	**B (SE)**
AoA	-5.767 (8.222)	-5.705 (8.183)	0.078 (0.279)	-4.741 (6.811)	-4.546 (6.742)	0.058 (0.387)
LoP	-4.848 (22.347)	-5.764 (22.211)	-0.270 (0.748)	-7.522 (18.514)	-11.125 (18.300)	-0.972 (1.038)
NoL	90.087 (83.423)	93.802 (83.410)	1.582 (2.807)	117.898 (69.113) (*[1])	117.830 (68.722) (*[2])	0.646 (3.896)
Age	-8.493 (5.074)	-8.627 (4.985)	-0.034 (0.169)	-3.357 (4.204)	-3.119 (4.107)	0.126 (0.235)
Sex (males = 0, females = 1)	-688.960 (120.902) ***	-701.664 (120.132) ***	-4.467 (4.101)	-482.311 (100.164) ***	-480.968 (98.978) ***	-0.793 (5.691)
Education	8.645 (36.726)	7.245 (36.621)	-0.925 (1.222)	-32.657 (30.427)	-20.992 (30.173)	2.366 (1.696)
Time interval	-	-	-1.621 (2.514)	-	-	-0.744 (3.489)
Constant	5637.488 (453.720) ***	5656.515 (462.512) ***	4.980 (17.024)	5054.783 (375.892) ***	4974.253 (381.069) ***	-21.129 (23.626)

(*[1])$p = 0.091$; (*[2])$p = 0.089$; *$p < 0.05$; **$p < 0.01$; ***$p < 0.001$.
Key: SA, surface area; IPL, inferior parietal lobule; AoA, age of acquisition; LoP, level of proficiency; NoL, number of actively spoken languages; ICV, intracranial volume; B, unstandardized coefficient B; SE, standard error.

Supplementary Table 28. Results for regression analyses for GMV in the IFG as dependent variable for bilinguals of the older subsample (n = 71) excluding ICV as predictor

	Left IFG			Right IFG		
	GMV at t1	GMV at t2	GMV difference	GMV at t1	GMV at t2	GMV difference
General model statistics	$R^2 = 0.082$<br>F[6, 64] = 0.947<br>p = 0.468	$R^2 = 0.084$<br>F[6, 64] = 0.984<br>p = 0.443	$R^2 = 0.070$<br>F[7, 63] = 0.682<br>p = 0.687	$R^2 = 0.101$<br>F[6, 64] = 1.202<br>p = 0.317	$R^2 = 0.082$<br>F[6, 64] = 0.957<br>p = 0.461	$R^2 = 0.136$<br>F[7, 63] = 1.413<br>p = 0.216
**Predictors**	**B (SE)**	**B (SE)**	**B (SE)**	**B (SE)**	**B (SE)**	**B (SE)**
AoA	5.124 (6.925)	8.740 (6.627)	0.890 (0.524) (*)	2.362 (8.254)	2.566 (8.099)	0.302 (0.396)
LoP	-4.630 (21.152)	-4.619 (20.163)	0.400 (1.617)	10.835 (25.211)	12.686 (24.642)	0.731 (1.220)
NoL	20.871 (79.152)	1.572 (75.217)	-4.842 (6.057)	-38.221 (94.339)	-14.503 (91.926)	6.496 (4.572)
Age	-12.525 (9.270)	-13.230 (8.841)	-0.289 (0.693)	-20.040 (11.048)	-17.997 (10.805)	-0.145 (0.523)
Sex (males = 0, females = 1)	-211.324 (124.461)	-168.058 (118.437)	7.775 (9.998)	-218.593 (148.342)	-195.727 (144.746)	7.772 (7.548)
Education	4.572 (34.096)	18.392 (32.341)	2.926 (2.553)	16.947 (40.638)	0.820 (39.525)	-2.628 (1.927)
Time interval	-	-	-1.895 (5.538)	-	-	-4.806 (4.181)
Constant	4819.675 (611.053) ***	4706.395 (616.880) ***	-25.586 (52.303)	5529.194 (728.297) ***	5448.568 (753.913) ***	3.923 (39.484)

(*)$p = 0.094$; *$p < 0.05$; **$p < 0.01$; ***$p < 0.001$.
Key: GMV, gray matter volume; IFG, inferior frontal gyrus; AoA, age of acquisition; LoP, level of proficiency; NoL, number of actively spoken languages; ICV, intracranial volume; B, unstandardized coefficient B; SE, standard error.

Supplementary Table 29. Results for regression analyses for GMV in the IPL as dependent variable for bilinguals of the older subsample (n = 71) excluding ICV as predictor

	Left IPL			Right IPL		
	GMV at t1	GMV at t2	GMV difference	GMV at t1	GMV at t2	GMV difference
General model statistics	$R^2 = 0.243$<br>$F[6, 64] = 3.430$<br>$p = 0.005$ **	$R^2 = 0.242$<br>$F[6, 64] = 3.410$<br>$p = 0.006$ **	$R^2 = 0.037$<br>$F[7, 63] = 0.345$<br>$p = 0.930$	$R^2 = 0.259$<br>$F[6, 64] = 3.721$<br>$p = 0.003$ **	$R^2 = 0.232$<br>$F[6, 64] = 3.220$<br>$p = 0.008$ **	$R^2 = 0.131$<br>$F[7, 63] = 1.355$<br>$p = 0.240$
**Predictors**	**B (SE)**	**B (SE)**	**B (SE)**	**B (SE)**	**B (SE)**	**B (SE)**
AoA	-40.075 (25.982)	-35.776 (24.759)	1.690 (1.759)	-14.368 (21.454)	-7.977 (21.364)	1.979 (1.307)
LoP	-8.622 (79.357)	2.066 (75.334)	4.853 (5.426)	-20.655 (65.527)	-16.422 (65.005)	2.953 (4.031)
NoL	272.592 (296.956)	246.732 (281.028)	-8.159 (20.330)	364.041 (245.204)	384.300 (242.495)	6.548 (15.103)
Age	-82.586 (34.778) *	-85.046 (33.033) *	-1.283 (2.325)	-73.015 (28.717) *	-76.628 (28.504) **	-1.786 (1.727)
Sex (males = 0, females = 1)	-1302.831 (466.943) **	-1205.904 (442.506) **	20.527 (33.561)	-1232.921 (385.567) **	-1075.851 (381.833) **	46.722 (24.931)
Education	-93.532 (127.920)	-70.136 (120.833)	4.297 (8.568)	-106.245 (105.627)	-83.781 (104.265)	6.368 (6.365)
Time interval	-	-	-6.899 (18.590)	-	-	-5.430 (13.810)
Constant	21008.182 (2292.500) ***	20845.002 (2304.806) ***	-68.225 (175.559)	18521.806 (1892.979) ***	18418.409 (1988.786) ***	-56.368 (130.417)

*$p < 0.05$; **$p < 0.01$; ***$p < 0.001$.
Key: GMV, gray matter volume; IPL, inferior parietal lobule; AoA, age of acquisition; LoP, level of proficiency; NoL, number of actively spoken languages; ICV, intracranial volume; B, unstandardized coefficient B; SE, standard error.

Supplementary Table 30. Results for regression analyses for CT in the IFG as dependent variable for bilinguals of the older subsample (n = 71) excluding ICV as predictor

	Left IFG			Right IFG		
	CT at t1	CT at t2	CT difference	CT at t1	CT at t2	CT difference
General model statistics	R^2 = 0.056<br>F[6, 64] = 0.638<br>p = 0.699	R^2 = 0.059<br>F[6, 64] = 0.669<br>p = 0.675	R^2 = 0.043<br>F[7, 63] = 0.407<br>p = 0.894	R^2 = 0.144<br>F[6, 64] = 1.789<br>p = 0.115	R^2 = 0.148<br>F[6, 64] = 1.852<br>p = 0.103	R^2 = 0.096<br>F[7, 63] = 0.954<br>p = 0.473
**Predictors**	**B (SE)**	**B (SE)**	**B (SE)**	**B (SE)**	**B (SE)**	**B (SE)**
AoA	-0.003 (0.002)	-0.002 (0.002)	< 0.001 (< 0.001)	0.002 (0.002)	0.002 (0.002)	< 0.001 (< 0.001)
LoP	0.001 (0.006)	0.004 (0.006)	0.001 (0.001)	0.005 (0.005)	0.008 (0.006)	0.001 (0.001)
NoL	0.017 (0.023)	0.006 (0.023)	-0.004 (0.003)	0.003 (0.019)	0.014 (0.021)	0.002 (0.003)
Age	< 0.001 (0.003)	< 0.001 (0.003)	< 0.001 (< 0.001)	-0.003 (0.002)	-0.003 (0.002)	< 0.001 (< 0.001)
Sex (males = 0, females = 1)	0.031 (0.036)	0.043 (0.036)	0.004 (0.005)	0.040 (0.029)	0.058 (0.033)	0.008 (0.006)
Education	0.003 (0.010)	0.006 (0.010)	0.001 (0.001)	0.019 (0.008) *	0.013 (0.009)	-0.001 (0.001)
Time interval	-	-	0.003 (0.003)	-	-	0.002 (0.003)
Constant	2.599 (0.179) ***	2.552 (0.187) ***	-0.025 (0.028)	2.543 (0.145) ***	2.551 (0.174) ***	-0.015 (0.029)

*$p < 0.05$; **$p < 0.01$; ***$p < 0.001$.
Key: CT, cortical thickness; IFG, inferior frontal gyrus; AoA, age of acquisition; LoP, level of proficiency; NoL, number of actively spoken languages; ICV, intracranial volume; B, unstandardized coefficient B; SE, standard error.

Supplementary Table 31. Results for regression analyses for CT in the IPL as dependent variable for bilinguals of the older subsample (n = 71) excluding ICV as predictor

	Left IPL			Right IPL		
	CT at t1	CT at t2	CT difference	CT at t1	CT at t2	CT difference
General model statistics	$R^2 = 0.183$<br>F[6, 64] = 2.389<br>p = 0.038 *	$R^2 = 0.208$<br>F[6, 64] = 2.802<br>p = 0.017 *	$R^2 = 0.053$<br>F[7, 63] = 0.505<br>p = 0.827	$R^2 = 0.170$<br>F[6, 64] = 2.179<br>p = 0.056	$R^2 = 0.185$<br>F[6, 64] = 2.420<br>p = 0.036 *	$R^2 = 0.054$<br>F[7, 63] = 0.518<br>p = 0.817
**Predictors**	**B (SE)**	**B (SE)**	**B (SE)**	**B (SE)**	**B (SE)**	**B (SE)**
AoA	-0.001 (0.002)	-0.001 (0.001)	< 0.001 (< 0.001)	< 0.001 (0.002)	< 0.001 (0.002)	< 0.001 (< 0.001)
LoP	-0.001 (0.005)	0.002 (0.005)	0.001 (0.001)	-0.003 (0.005)	< 0.001 (0.005)	0.001 (0.001)
NoL	0.028 (0.017)	0.020 (0.017)	-0.003 (0.003)	0.033 (0.019) (*[1])	0.033 (0.018) (*[2])	-0.001 (0.004)
Age	-0.005 (0.002) *	-0.006 (0.002) **	< 0.001 (< 0.001)	-0.006 (0.002) **	-0.006 (0.002) **	< 0.001 (< 0.001)
Sex (males = 0, females = 1)	0.049 (0.027)	0.061 (0.027) *	0.005 (0.005)	-0.029 (0.030)	-0.003 (0.028)	0.008 (0.006)
Education	< 0.001 (0.007)	0.003 (0.007)	0.001 (0.001)	0.005 (0.008)	0.005 (0.008)	< 0.001 (0.002)
Time interval	-	-	0.002 (0.003)	-	-	0.001 (0.003)
Constant	2.845 (0.134) ***	2.851 (0.139) ***	-0.013 (0.027)	2.883 (0.149) ***	2.861 (0.147) ***	- 0.015 (0.031)

(*[1])*p* = 0.093; (*[2])*p* = 0.068; **p* < 0.05; ***p* < 0.01; ****p* < 0.001.
Key: CT, cortical thickness; IPL, inferior parietal lobule; AoA, age of acquisition; LoP, level of proficiency; NoL, number of actively spoken languages; ICV, intracranial volume; B, unstandardized coefficient B; SE, standard error.

Supplementary Table 32. Results for regression analyses for SA in the IFG as dependent variable for bilinguals of the older subsample (n = 71) excluding ICV as predictor

	Left IFG			Right IFG		
	SA at t1	SA at t2	SA difference	SA at t1	SA at t2	SA difference
General model statistics	$R^2 = 0.160$<br>F[6, 64] = 2.026<br>p = 0.075	$R^2 = 0.163$<br>F[6, 64] = 2.077<br>p = 0.068	$R^2 = 0.207$<br>F[7, 63] = 2.350<br>p = 0.034 *	$R^2 = 0.108$<br>F[6, 64] = 1.289<br>p = 0.275	$R^2 = 0.110$<br>F[6, 64] = 1.319<br>p = 0.262	$R^2 = 0.052$<br>F[7, 63] = 0.494<br>p = 0.835
**Predictors**	**B (SE)**	**B (SE)**	**B (SE)**	**B (SE)**	**B (SE)**	**B (SE)**
AoA	3.386 (2.085)	4.261 (2.080) *	0.288 (0.118) *	-0.623 (2.576)	-0.844 (2.542)	0.010 (0.179)
LoP	-3.912 (6.369)	-4.729 (6.328)	-0.411 (0.363)	-0.015 (7.868)	-0.785 (7.735)	-0.483 (0.553)
NoL	-6.775 (23.833)	-7.021 (23.606)	1.037 (1.360)	-19.410 (29.441)	-20.351 (28.855)	-0.149 (2.073)
Age	-4.578 (2.791)	-4.931 (2.775)	-0.111 (0.156)	-4.008 (3.448)	-3.326 (3.392)	-0.047 (0.237)
Sex (males = 0, females = 1)	-91.784 (37.476) *	-82.786 (37.171) *	-0.381 (2.244)	-92.770 (46.295) *	-97.974 (45.435) *	-2.927 (3.422)
Education	4.878 (10.267)	6.040 (10.150)	-0.077 (0.573)	-5.244 (12.682)	-4.411 (12.407)	0.585 (0.874)
Time interval	-	-	-3.791 (1.243) **	-	-	-2.696 (1.895)
Constant	1550.417 (183.991) ***	1556.333 (193.604) ***	14.891 (11.740)	1802.968 (227.288) ***	1760.451 (236.652) ***	9.217 (17.900)

*$p < 0.05$; **$p < 0.01$; ***$p < 0.001$.

Key: SA, surface area; IFG, inferior frontal gyrus; AoA, age of acquisition; LoP, level of proficiency; NoL, number of actively spoken languages; ICV, intracranial volume; B, unstandardized coefficient B; SE, standard error.

Supplementary Table 33. Results for regression analyses for SA in the IPL as dependent variable for bilinguals of the older subsample (n = 71) excluding ICV as predictor

	Left IPL			Right IPL		
	SA at t1	SA at t2	SA difference	SA at t1	SA at t2	SA difference
General model statistics	$R^2 = 0.261$ F[6, 64] = 3.775 p = 0.003 **	$R^2 = 0.265$ F[6, 64] = 3.854 p = 0.002 **	$R^2 = 0.034$ F[7, 63] = 0.320 p = 0.942	$R^2 = 0.153$ F[6, 64] = 1.928 p = 0.090	$R^2 = 0.151$ F[6, 64] = 1.894 p = 0.095	$R^2 = 0.042$ F[7, 63] = 0.396 p = 0.901
**Predictors**	**B (SE)**	**B (SE)**	**B (SE)**	**B (SE)**	**B (SE)**	**B (SE)**
AoA	-12.485 (9.043)	-12.180 (8.918)	0.109 (0.311)	-6.470 (8.074)	-5.896 (7.968)	0.154 (0.507)
LoP	-8.928 (27.620)	-9.483 (27.136)	-0.011 (0.960)	-5.904 (24.661)	-10.182 (24.245)	-1.251 (1.566)
NoL	63.219 (103.354)	70.337 (101.228)	2.405 (3.598)	74.720 (92.282)	73.301 (90.442)	0.438 (5.866)
Age	-18.073 (12.104)	-18.576 (11.899)	-0.325 (0.412)	-12.126 (10.808)	-12.499 (10.631)	-0.133 (0.671)
Sex (males = 0, females = 1)	-600.201 (162.517) ***	-601.420 (159.394) ***	-2.132 (5.939)	-388.319 (145.107) **	-397.231 (142.410) **	-3.723 (9.683)
Education	-13.184 (44.522)	-9.806 (43.525)	-0.418 (1.516)	-52.052 (39.752)	-38.940 (38.887)	3.325 (2.472)
Time interval	-	-	-3.731 (3.290)	-	-	-1.935 (5.364)
Constant	6613.551 (797.892) ***	6636.314 (830.206) ***	22.187 (31.069)	5845.656 (712.415) ***	5832.205 (741.749) ***	-5.425 (50.655)

*$p < 0.05$; **$p < 0.01$; ***$p < 0.001$.

Key: SA, surface area; IPL, inferior parietal lobule; AoA, age of acquisition; LoP, level of proficiency; NoL, number of actively spoken languages; ICV, intracranial volume; B, unstandardized coefficient B; SE, standard error.
